# Delayed differentiation of epidermal cells walls can underlie pedomorphosis in plants: the case of pedomorphic petals in the hummingbird-pollinated *Caiophora hibiscifolia* (Loasaceae, subfam. Loasoideae) species

**DOI:** 10.1186/s13227-021-00186-x

**Published:** 2022-01-03

**Authors:** Marina M. Strelin, Eduardo E. Zattara, Kristian Ullrich, Mareike Schallenberg-Rüdinger, Stefan Rensing

**Affiliations:** 1 Grupo de Investigación en Ecología de la Polinización, Laboratorio Ecotono, INIBIOMA (CONICET - Universidad Nacional del Comahue), San Carlos de Bariloche, Río Negro Argentina; 2 Department of Evolutionary Biology, August Thienemann Str. 2, 24306 Plön, Germany; 3 IZMB - Institut für Zelluläre und Molekulare Botanik, Abt. Molekulare Evolution, University of Bonn, Kirschallee 1, 53115 Bonn, Germany; 4 Plant Cell Biology, Department of Biology, University of Marburg, Marburg, Germany

**Keywords:** Heterochrony, Pedomorphosis, Cell shape, Epidermis differentiation, Flower evolution, Petal, Transcriptome, Loasoideae, Pollination

## Abstract

**Background:**

Understanding the relationship between macroevolutionary diversity and variation in organism development is an important goal of evolutionary biology. Variation in the morphology of several plant and animal lineages is attributed to pedomorphosis, a case of heterochrony, where an ancestral juvenile shape is retained in an adult descendant. Pedomorphosis facilitated morphological adaptation in different plant lineages, but its cellular and molecular basis needs further exploration. Plant development differs from animal development in that cells are enclosed by cell walls and do not migrate. Moreover, in many plant lineages, the differentiated epidermis of leaves, and leaf-derived structures, such as petals, limits organ growth. We, therefore, proposed that pedomorphosis in leaves, and in leaf-derived structures, results from delayed differentiation of epidermal cells with respect to reproductive maturity. This idea was explored for petal evolution, given the importance of corolla morphology for angiosperm reproductive success.

**Results:**

By comparing cell morphology and transcriptional profiles between 5 mm flower buds and mature flowers of an entomophile and an ornitophile Loasoideae species (a lineage that experienced transitions from bee- to hummingbird-pollination), we show that evolution of pedomorphic petals of the ornithophile species likely involved delayed differentiation of epidermal cells with respect to flower maturity. We also found that developmental mechanisms other than pedomorphosis might have contributed to evolution of corolla morphology.

**Conclusions:**

Our results highlight a need for considering alternatives to the flower-centric perspective when studying the origin of variation in flower morphology, as this can be generated by developmental processes that are also shared with leaves.

**Graphical Abstract:**

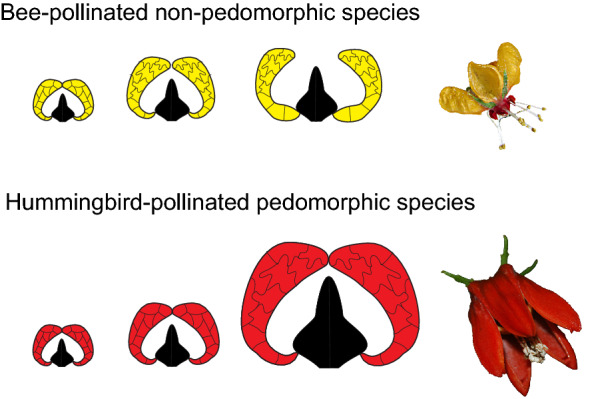

**Supplementary Information:**

The online version contains supplementary material available at 10.1186/s13227-021-00186-x.

## Background

An intriguing issue in evolutionary biology is the relationship between macroevolutionary patterns of diversity and the origin of variation in organism development. In this context, attention has been paid to the role of heterochrony in the origin of the morphological variation. Heterochronic variation in the observable morphology of organisms is generated by alteration of developmental trajectories involving shifts in the timing of somatic differentiation relative to the timing of reproductive maturity [1]. This could result either in ‘overdeveloped’ somatic morphologies, i.e., peramorphosis, or in ‘underdeveloped’ somatic morphologies, i.e., pedomorphosis in the descendant lineage [3]. Heterochronic variation fueled adaptation to changing selection environments in an array of animal and plant lineages [23, 28, 33] but its cellular and molecular basis remains poorly understood, particularly in plants [5].

Plant development differs qualitatively from animal development in that cells are enclosed by cell walls. Plant cells divide, expand and change their shape during development but do generally not migrate. Moreover, the growth of plant organs is physically limited by the epidermis [8]. Hence, any change to the timing of differentiation of the epidermis, when compared to reproductive maturity, should yield heterochronic variation in the morphology of plant organs. When defining reproductive maturity in plants, it is important to keep in mind that plants are modular organisms. Reproductive maturity can, therefore, be defined at the whole plant level, e.g., inflorescence initiation, but also for separate plant reproductive modules, e.g., anther dehiscence and stigma receptivity in a flower.

In many plant organs, such as cotyledons, normal leaves and petals (leaf-derived structures), the surface of the epidermis is composed to a large extent by closely packed jigsaw puzzle-shaped pavement cells [24, 26, 28]. Similar cells are also common in the leaf epidermis of ferns and gymnosperms [42]. They develop from rectangular or polygonal protodermal cells that become lobed as they grow [9, 27]. Cell differentiation into the jigsaw puzzle-shaped cell type is not homogeneous along the leaf blade. For instance, in *Arabidopsis thaliana* and in *Cardamine hirsuta*, differentiation into puzzle-shaped pavement cells is basipetal, i.e., the first cells to undergo differentiation into the lobed shape are at the apex of the leaf blade, while the last ones are at the base of the leaf blade [18]. In differentiated plant organs, lobed cell shapes appear to be important for the correct spacing of other epidermal cell types, such as stomata and trichomes, for increasing the stability of the epidermis, which is often under considerable tension from internal cells, and for resisting the mechanical stresses the cell walls encounter due to turgor pressure: if the cells had regular polygonal shapes, large cells would bulge out excessively under turgor pressure and burst (works cited in [30]).

Acquisition of lobed cell walls during plant organ differentiation decelerates cell growth. This happens, because cell wall lobeyness, defined as the tendency to present lobes (convex areas) and indentations (concave areas) [30], decreases turgor pressure on the anticlinal cell walls (those perpendicular to the organ surface). This cell dynamic can in turn decelerate tissue growth [19]. We propose that changes in the timing of cell wall lobeyness development with respect to reproductive maturity might underlie morphological heterochrony in leaves and in leaf-derived structures. Peramorphosis takes place when cell lobeyness starts developing at an earlier stage relative to reproductive maturity in a descendant lineage. This results in a descendant morphology that is small, but ‘overdeveloped’ in shape. On the other hand, pedomorphosis takes place when cell lobeyness starts developing at a later stage relative to reproductive maturity. This results in a prolongation of juvenile growth patterns and a descendant morphology that is large, but juvenile in shape [13].

Two molecular mechanisms have been proposed to increase cell wall lobeyness during plant organ differentiation. According to the classical perspective [9], differential deposition of cellulose fibres and microtubule bundles on the anticlinal cell walls results in heterogeneous resistance to turgor pressure along these walls. Interplay between turgor pressure and anticlinal cell wall properties results in outgrowth of the less resistant sections of the anticlinal cell wall. In turn, recruitment of actin filaments in expanding lobes reinforces lobe outgrowth. Kinesin-like proteins, actin-related proteins, Rac-like GTP-binding proteins, Rho of plants (ROP) proteins, CRIB domain-containing proteins, a cellulose synthase and a CLIP-associated protein participate in this lobe-formation mechanism [27, 30]. There is also controversial evidence that auxin-related proteins ABP1 and PIN participate in this mechanism of cell morphogenesis [30]. This molecular mechanism of cell wall lobeyness appears to be common to all monocots and eudicots [42]. According to an alternative perspective [9], demethylesterification of pectin nanofilaments, along and across the anticlinal cell wall, results in local swelling of pectin nanofilaments, thus causing local anticlinal cell wall expansion. Under this scenario, local cell wall expansion does not depend on an interaction between cell wall properties and turgor pressure but on properties that are intrinsic to the anticlinal cell wall. Pectin methylesterases and pectinesterase inhibitors participate in this mechanism of lobe formation [17]. Galacturonosyltransferases also participate in pectin biosynthesis and are related to cell wall lobeyness [27].

Here we explored the idea that changes in the timing of cell wall lobeyness development generate heterochronic variation in the morphology of plant organs, by focusing on the angiosperm flower. We chose to focus on this plant module given its importance for angiosperm reproductive success. The evolutionary versatility of flower traits is frequently related to different pollination and reproductive modes, and is among the most important drivers of angiosperm speciation and diversification [40]. Corolla morphology is particularly important to plant–pollinator interactions, as it determines the location on which pollen is placed and picked up from the pollinator body [11]. Heterochrony was involved in adaptation of corolla morphology to different pollinators in lineages as diverse as *Delphinium* (Ranunculaceae) [14], *Aquilegia* (Ranunculaceae) [28], *Calceolaria* (Calceolariaceae) [38] and *Mimulus* (Phyrmaceae) [15].

**Fig. 1:**
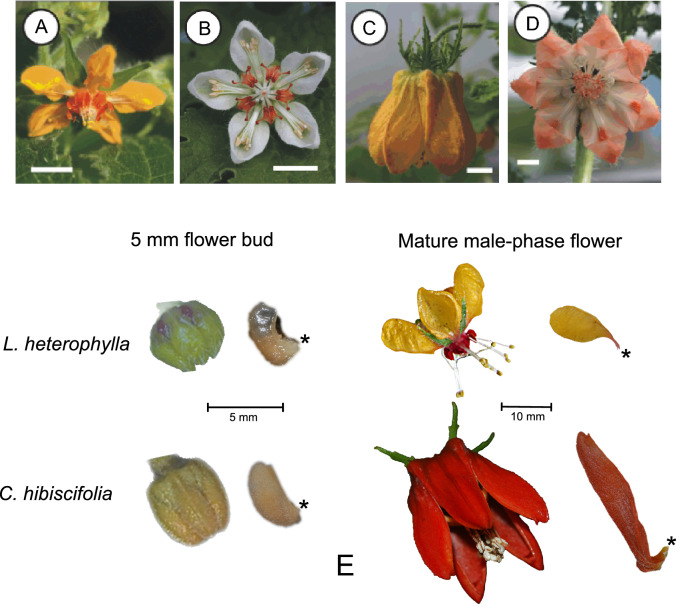
Mature flowers of two bee-pollinated Loasoideae species, *Loasa acerifolia* (**A**) and *Caiophora cernua* (**B**), and two hummingbird-pollinated Loasoideae species, *Caiophora carduifolia* (**C**) and *Caiophora chuquitensis* (**D**). Scale bar = 10 mm. 5 mm flower buds and male-phase flowers of *L. heterophylla* and *C. hibiscifolia*, the bee- and the hummingbird-pollinated species sampled for this study. The petal base is indicated with ‘*’ (**E**). Images ‘**A**’ to ‘**D**’ were taken from Strelin et al. [36]

**Fig. 2:**
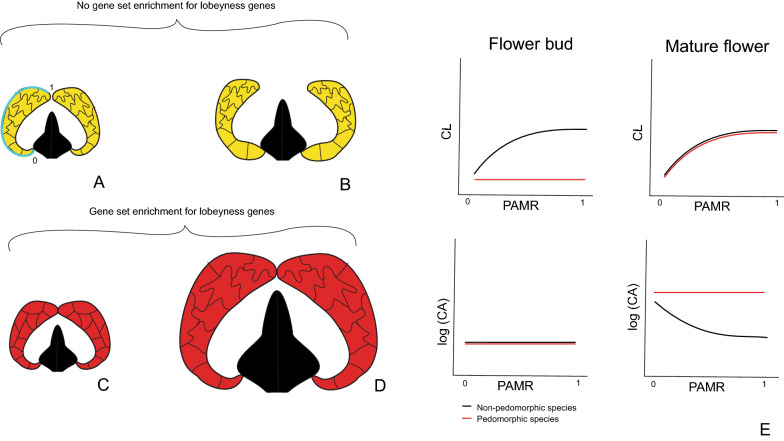
Graphical representation of the predictions of the hypothesis that delayed differentiation of petal epidermal cells, when compared to flower reproductive maturity, results in a pedomorphic corolla. Cell wall lobeyness and cell area in flower buds of a fixed size (**A**, **C**) and in mature flowers (**B**, **D**) of a non-paedomorphic species (**A**, **B**) and a pedomorphic species (**C**, **D**) are represented. Enrichment of gene sets related to cell wall lobeyness is expected in the pedomorphic species (**A**–**D**). Predicted relationships between cell wall lobeyness (CL) and position along the petal midrib (PAMR) and between cell area, *log*(CA), and PAMR are represented for the buds and mature flowers of the non-pedomorphic and the pedomorphic species (**E**). The petal midrib is drawn in cyan. The most basal position along the petal midrib is indicated with 0; the most apical position is indicated with 1. Notice that lobed cell walls decelerate cell and tissue growth (except for the petal base, which has regular and expanding cells). Petal shape differentiation into a structure with a disproportionally expanded petal base drives corolla opening and exposure of fertile flower structures. Cell wall lobeyness appears early in the non-pedomorphic species. Hence, the mature flowers of the non-pedomorphic species are small but open. Instead, cell wall lobeyness appears later in the pedomorphic species. Hence, the petal continues growing at bud-like rates until a more advanced stage. As cell walls remain regular along the whole middle rib until later, mature flowers in the pedomorphic species are large but their shape resembles that of a flower bud (expansion of the petal base and flower opening are not completed at maturity)

The Loasaceae subfam. Loasoideae (Cornales) is an interesting study system to dip inside the cellular and molecular mechanisms behind morphological heterochrony. Most of the Loasoideae species are bee-pollinated and present small and open corollas; ancestral reconstructions of flower morphology and its pollination syndrome suggest that bee-pollination (entomophily) and small open corollas represent the ancestral condition in this subfamily [34] (Additional file 1). Hummingbird pollination (ornithophily) emerged at least twice during evolution of Loasoideae: two of the most diverse Loasoideae genera, *Caiophora* C. Presl and *Nasa* Weigend include hummingbird pollinated species [2, 34]. The corolla of hummingbird-pollinated Loasoideae species is large. As the expansion of the petal base that results in flower opening is not completed, the corolla of hummingbird-pollinated species is narrower than in bee-pollinated species (Fig. 1) [35, 37]. We propose that the evolution of large flowers with an unexpanded petal base in hummingbird-pollinated Loasoideae species resulted from delayed development of basipetal cell wall lobeyness (Fig. 2). Instead, in non-pedomorphic bee-pollinated species, earlier basipetal cell differentiation results in disproportionate expansion of the petal base and small flower size (Fig. 2). Under this hypothesis, we expect an enrichment of a first lobeyness gene set putatively driving interactions between turgor pressure and cell wall properties, and/or of a second lobeyness gene set related to intrinsic properties of the cell wall, when comparing mature flowers to pre-anthesis buds in the pedomorphic, hummingbird-pollinated species (Fig. 2). Since in bee-pollinated species cell wall lobeyness develops earlier than in pre-anthesis buds, we do not expect enrichment of these gene sets among the two stages in these species (Fig. 2).

Here we present data about cell morphology (lobeyness, area and length-to-width ratio) and RNAseq transcriptional profiles from petals of two Loasoideae species (*Loasa heterophylla* Hook. & Arn. and *Caiophora hibiscifolia* (Griseb.) Urb. & Gilg.). These differ in pollination strategy and in corolla shape during flower anthesis (Fig. 2). *L. heterophylla* is a bee-pollinated species with open, small, corollas; *C. hibiscifolia* is pollinated by hummingbirds, and presents narrow and large corollas [6, 34]. Based on phylogenetic reconstruction (Additional file 1), we assumed *L. heterophylla* to be representative of the ancestral condition in Loasoideae and asked whether petal pedomorphosis in *C. hibiscifolia* is paralleled by (1) delayed development of basipetal cell wall lobeyness and (2) enrichment of gene sets related to cell wall lobeyness, when comparing 5 mm flower buds to mature flowers (Fig. 2).

In the plant evo-devo literature, flower organ differentiation and maturation have been related to flower MADS-box homeotic genes, and to down-stream associated genes that affect organ boundary formation and growth [7, 10]. We, therefore, tested whether genes specifically related to flower differentiation and maturation in the literature were also enriched in the *C. hibiscifolia* comparison. Moreover, we explored whether other developmental mechanisms, besides pedomorphosis, could account for interspecific differences in corolla morphology in Loasoideae. Since flower opening takes place by cell expansion, but also by cell elongation at the petal base [41], we asked whether cell elongation was more pronounced at the petal base in *L. heterophylla*, when compared to *C. hibiscifolia*. We also asked whether the flower buds vs. mature flowers comparison in *L. heterophylla* was enriched in genes related to cell elongation in the literature [25, 41]. Finally, since increased initial cell number or increased cell proliferation can also contribute to the evolution of large flowers [20, 39], we asked to what extent these two processes contributed to large flower size in *C. hibiscifolia*.

## Materials and methods

### Study system

Loasaceae subfam. Loasoideae is a monophyletic and mostly Neotropical angiosperm subfamily. South Andean loasas diverged from the core Loasoideae during the mid-Eocene, around 45 Ma. Within South-Andean loasas, *Blumenbachia* Schrad., *Loasa* Adans., *Pinnasa* Weigend & R.H and *Scyphanthus* Sweet are entomophile, bee-pollinated genera. Divergence of these genera took place between the late Eocene, around 40 Ma., and the middle Oligocene, around 26 Ma. The South-Andean loasas genus *Caiophora* C. Presl. originated during the middle Oligocene, and several speciation events took place during the last 12 Ma. [6]. Remarkable speciation in the genus has been attributed to different Andean uplift events [6, 36] and to the acquisition of hummingbird (ornithophily) and rodent (therophily) pollination at high-elevation habitats [36]. Loasoideae flowers are protandric and their shape is highly complex. They present a corolla with separate pouch-shaped petals protecting the stamens and a whorl of androecium derived nectar scales [4]. Flowers are small and pendulous in bee-pollinated species and require the pollinator to land and hold onto the flower by grappling the nectar scales. Bee-pollinated flowers present open corollas, sometimes with highly reflexed petals, which make the nectar scales visible and easy to grasp (Fig. 1). Petal development in bee-pollinated species involves progressive expansion of the petal base [35, 37]. This shape change results in an open corolla with visible nectar scales. Expansion of the petal base is not completed in hummingbird-pollinated species and results in a narrow corolla [35, 37] (Fig. 1). Narrow corollas in hummingbird-pollinated species ensure that the body of the hovering hummingbird contacts the fertile flower structures [34]. Reversion from hummingbird- to bee-pollination took place at least once in *Caiophora*, and was paralleled by the evolution of small and open corollas with an expanded petal base [36] (Fig. 1).

This study includes the sampling of two species (Fig. 1). *L. heterophylla* originated around 15 Ma., is bee-pollinated and presents a small and open corolla composed of remarkably reflexed petals; *C. hibiscifolia* originated during the last 5 Ma., is pollinated by hummingbirds, and presents narrow and large corollas [6, 34]. The lineages giving rise to these two species diverged from a common ancestor around 40 Ma. [6]. We assumed that the pollination strategy and the corolla morphology of *L. heterophylla* represent the ancestral conditions in Loasoideae, based on maximum likelihood and Bayesian ancestral character state reconstructions (Additional file 1). *L. heterophylla* is distributed in low-elevation habitats in southern Chile; *C. hibiscifolia* is distributed in middle to high elevation habitats in the NW of Argentina [36].

### Sampling

Sampling was conducted at the Botanical Gardens of Bonn University during the summer of 2013, except for two mature flower samples and a flower bud sample of *L. heterophylla*, which were collected during the summer of 2012. Our choice of species was constrained by availability of enough individual plants at the gardens. We sampled three outdoors individuals of *L. heterophylla* and three outdoors individuals of *C. hibiscifolia*. One mature male-phase flower and one flower bud with a diameter of 5 mm were sampled from each individual (this makes a total of 12 samples, including mature flowers and flower buds). One petal was collected from each flower and flower bud and stored in liquid air for RNA extraction. RNA sampling included an additional mature flower of *L. heterophylla* (making a total of 13 samples). An additional petal was collected from the mature flower and from the flower bud of one individual of each species and stored in liquid air for electron microscope imaging (a total of four samples).

### SEM imaging

Scanning electron microscope (SEM) photos were obtained from the inner (concave) side of the petal, as the outer surface of the petal is covered with trichomes and the epidermal cells are not clearly visible. Before imaging, petals were critical-point-dried after fixation in 70% ethanol + 4% formaldehyde for at least 24 h and dehydrated with ethanol and acetone. After critical-point-drying (CP drying) petals were mounted as flat as possible on SEM holders. Dried samples were sputter-coated with a thin layer (< 30 nm) of palladium (Junker Edelmetalle, Waldbüttelbrunn, Germany). Scanning electron microscopy was performed with a LEO 1450 SEM (Zeiss, Jena, Germany). Images were recorded with a digital image acquisition system DISS 5 (Point Electronic, Halle, Germany). Petals were cut along the middle to reveal epidermal cells from the region adjacent to the petal midrib, and 8–9 pictures were obtained between the apical and basal end ends of the petal with a magnification of 100×. The first and the last picture in each sequence always corresponded to the apical and basal ends. Positions of the images along the petals were standardized across samples, so that a position at 0 corresponded to the basal end and a position of 1 corresponded to apical end. The SEM stage was tilted with the samples 30° to obtain good contrast, and the resulting distortion was compensated with the SEM-function ‘Tilt correction’.

### Petal and cell geometry measurements

We randomly chose 10 cells from each SEM image and measured cell area (CA) in squared micrometers, and cell lobeyness. Cell lobeyness was calculated using a solidity index *S* ranging between 0 and 1. Cells with regular walls have *S* values that are closer to 1; cells with lobed walls have *S* values that are closer to 0. Cell lobeyness (CL) was calculated as 1 − *S*, so that cells with regular walls have CL values that are closer to 0, while cells with lobed walls have CL values closer to 1. We calculated the cell length-to-width ratio (CLWR) in the same longitudinal orientation of the petal, by measuring the maximum cell length in the longitudinal orientation of the petal and the maximum cell width in the perpendicular direction, and dividing the first by the second.

Pictures of the two halves of the cut petals used for SEM images were made to measure total petal area. Total petal area in squared micrometers was obtained by adding up the areas of the two cut petal halves. We obtained an estimate of the number of epidermal cells in the petals of the flower bud and the mature flower of each species by calculating the area of each SEM image, counting the number of epidermal cells in each SEM image, obtaining the average number of cells per squared micrometer in each SEM image and multiplying it by total petal area. We, therefore, have 8–9 calculations of cell number per petal, each corresponding to a different SEM image. We obtained a final average cell number per petal by averaging these calculations and also obtained the standard error of this estimated value. Cells were measured and counted with Image J [31].

### Statistical analyses of CL, CA and CLWR along the middle rib of the petal

We used a linear model regression, including a quadratic term, on the relationship between cell lobeyness (CL) and the position along the petal midrib (PAMR) of the SEM image. We also used a linear model regression, including a quadratic term, on the relationship between the logarithm of cell area (CA), from here on *log*(CA), and PAMR. These models were used to test predictions about cell morphology along the petal midrib in *L. heterophylla* and *C. hibiscifolia*, under a scenario of delayed differentiation of epidermal cells in the later (Fig. 2). A linear model including a quadratic term was also used on the relationship between cell length-to-width ratio (CLWR) and PAMR. This last model was used to test whether differences in cell elongation at the petal base are related to interspecific differences in the expansion of the petal base. Since the relationship between CL, *log*(CA) and CLWR and PAMR was expected to differ between developmental stages and species, and we also expected the effect of the interaction between PAMR and developmental stage to depend on the species (Fig. 2), our models included a triple interaction among PAMR, developmental stage and species.

### RNA preparation and sequencing

Frozen petal samples were pulverized with a mortar and pestle and total RNA was extracted and purified using the RNeasy Plant Mini Kit (Qiagen). Total RNA was checked for integrity using a BioAnalyzer with an Agilent RNA 6000 Nano Chip, following the manufacturer’s instructions (Agilent, https://www.agilent.com). We provided 5 μg of total cDNA from each sample to the Max Plank Genome Center Cologne for sequencing. cDNA was sequenced as 100 bp, paired-end reads using HiSeq2500 (Illumina). Libraries obtained for two out of the three *L. heterophylla* bud samples were sequenced later, at 150 bp. All data are available through NCBI’s Short Read Archive (BioProject accession: PRJNA763894).

### De novo transcriptome assembly, read mapping and fragment counting

FastQ files for each species were quality trimmed with Trimmomatic (http://www.usadellab.org/cms/?page=trimmomatic), and fed to Trinity-v2.4.0 (https://github.com/trinityrnaseq/trinityrnaseq/wiki) to assemble species-specific reference transcriptomes. Completeness of each assembly was assessed using BUSCO (https://busco.ezlab.org) v4.1.3 with the embryophyta_odb10 lineage data set. Mapping of reads from each species to its assembled reference transcriptome and transcript abundance estimation was performed using the *align_and_estimate_abundance.pl* and *abundance_estimates_to_matrix.pl* perl scripts included in Trinity-v2.4.0 [16], using Bowtie2 [21] as alignment method and RSEM [22] as abundance estimation method.

### Reference genome gene model annotation and transcriptome-to-genome mapping

Since there is no available genome from any species within the family Loasaceae, available genomes from the closest possible families were considered as a source for annotated “common” reference. The genome from the Chinese Happy Tree, *Camptotheca acuminata* (Nyssaceae) was chosen for this purpose (https://doi.org/10.1093/gigascience/gix065). The gene models were annotated using Trinotate (https://github.com/Trinotate/Trinotate.github.io/wiki). Details about gene model annotation can be found in the R Report of the RNAseq analysis (Additional file 2). *L. heterophylla* and *C. hibiscifolia* transcripts were mapped to *C. acuminata* gene models using BLASTx searches. Based on these mappings, a mapping table assigning a *C. acuminata* gene model to isoforms of each species that had a hit with an *e* value of 10e−3 or lower was built; all other isoforms were discarded.

### Merging of count tables and exploratory analyses

Using the above mapping tables, a *C. acuminata* gene model was assigned to each isoform (row) of the read count table of each the two sequenced species. Reads for multiple isoforms matching a single *C. acuminata* gene model were collapsed by summing, while isoforms without a match were discarded. The resulting tables were then merged into a single table using *C. acuminata* gene model id as the key field. Using an inner join, only collections of transcripts with hits to *C. acuminata* gene models in both species were retained. Count data was normalized using a weighted trimmed mean of the *log* expression ratios (TMM, [29]), which normalizes by effective library size, but not by feature length. Then, counts were normalized to counts-per-million (cpm) using the *cpm* function (edgeR library). A filter was applied to remove any genes not having at least 1 cpm in at least three samples. We performed clustering and PCA exploratory analyses to check that samples clustered according to our sampling design, based on their RNA expression profile.

### Differential gene expression analysis

Differentially expressed genes (DEGs) were detected using the DESeq2 (https://doi.org/10.1186/s13059-014-0550-8) approach, which estimates variance-mean dependence in count data and tests for DEGs using a model based on the negative binomial distribution. Four contrasts were created for DEGs: (1) *L. heterophylla* 5 mm bud vs*. L. heterophylla* mature flower; (2) *C. hibiscifolia* 5 mm bud vs*. C. hibiscifolia* mature flower; (3) *L. heterophylla* 5 mm bud vs. *C. hibiscifolia* 5 mm bud; (4) *L. heterophylla* mature flower vs. *C. hibiscifolia* 5 mm mature flower*.* The false discovery rate (FDR) threshold for significance was set to 0.05. Heatmaps of DEGs for *L. heterophylla* and *C. hibiscifolia* samples can be consulted in the R report (Additional file 2).

### Gene set enrichment analysis (GSEA)

We conducted gene set enrichment analyses (GSEAs) for genes that are related to cell wall lobeyness in each of the four contrasts described above. Each GSEA requires a ranked list of the DEGs in the contrast. Were, therefore, ranked the differentially expressed genes in our four contrasts, based on their *log2*(fold-change). GSEA also requires a signature or an array of signatures that define a set of focal genes, cell lobeyness and cell elongation genes in our case. We created two signatures of cell wall lobeyness. The first includes genes that affect cell wall lobeyness trough interaction between turgor pressure and heterogeneous deposition of cellulose fibres and microtubule bundles on anticlinal cell walls, plus lobe outgrowth driven by actin filaments [27, 30]. We call this the ‘turgor pressure-cell wall interaction’ (TCWI) signature. The second signature includes genes that affect cell wall lobeyness through processes that are intrinsic to the cell wall and involve heterogeneous pectin demethylesterification of anticlinal cell walls [17]. We call this the ‘intrinsic cell wall property’ (ICWP) signature. The TCWI signature includes genes that code for kinesin-like proteins, actin-related proteins, Rac-like GTP-binding proteins, Rho of plants (ROP) proteins, CRIB domain-containing proteins, a cellulose synthase and a CLIP-associated protein. It also includes genes that code for the auxin-related proteins ABP1 and PIN. The ICWP signature includes genes that code for pectin methylesterases and pectinesterase inhibitors, as well as galacturonosyltransferases. We created an additional signature for genes that are related to differentiation and maturation of flower organs in the literature. These include genes coding for MADS-box proteins, for BEL1-like homeodomain proteins, for the Zinc finger protein JAGGED, for Auxin response factor ETTIN, for DELLA protein RGA, for protein SPOROCYTELESS and for TCP transcription factors [7, 10]. We call this the ‘flower differentiation-maturation’ (FDM) signature. We created a final signature for genes that are related to cell elongation in the literature. This included genes coding for DELLA proteins [41] and aquaporin genes [25, 41]. We call this the ‘cell elongation’ (CE) signature. To create the signatures, we first searched among the GO terms of the annotated *C. acuminata* reference transcriptome for protein and gene names in the protein families described above, as they appear in UniProt (https://www.uniprot.org/). The gene IDs of the filtered genes were kept and used in the GSEA. We did four GSEA analyses, one for each contrast. In each GSEA we looked for matches between the gene IDs of the TCWI, the ICWP, the FDM and the CE signatures and the gene IDs of the ranked DEGs list of the contrast. GSEA was done using the *GSEA* function of the clusterProfiler R library [43]. This function calculates a sum statistic for each signature and an enrichment score. It also permutes the rows of the ranked DEGs table, to calculate a null distribution and a *P* value for the enrichment score.

## Results

### Cell geometry and cell number in petals of *L. heterophylla* and *C. hibiscifolia*

Triple interaction terms between PAMR (position along the petal midrib) developmental stage and species were significant in the regressions describing the patterning of CL (cell lobeyness), *log*(CA) (logarithm of cell area) and CLWR (cell length–width ratio) along the petal midrib (*t* = 3.806, *P* < 0.0005; *t* = 3.347, *P* < 0.0005; *t* = 3.685, *P* < 0.0005 for interaction with the linear term; *t* = − 3.365, *P* < 0.0005; *t* = − 4.048, *P* < 0.0001; *t* = − 2.880, *P* < 0.005 for interaction with the quadratic term). This means that the relationship between CL, *log*(CA) and CLWR and PAMR differed between developmental stages and species, and that developmental changes of CL, *log*(CA) and CLWR along the petal midrib also differed between species. In the results reported below, the 5 mm flower bud and *L. heterophylla* are always the basal factor levels.

**Fig. 3:**
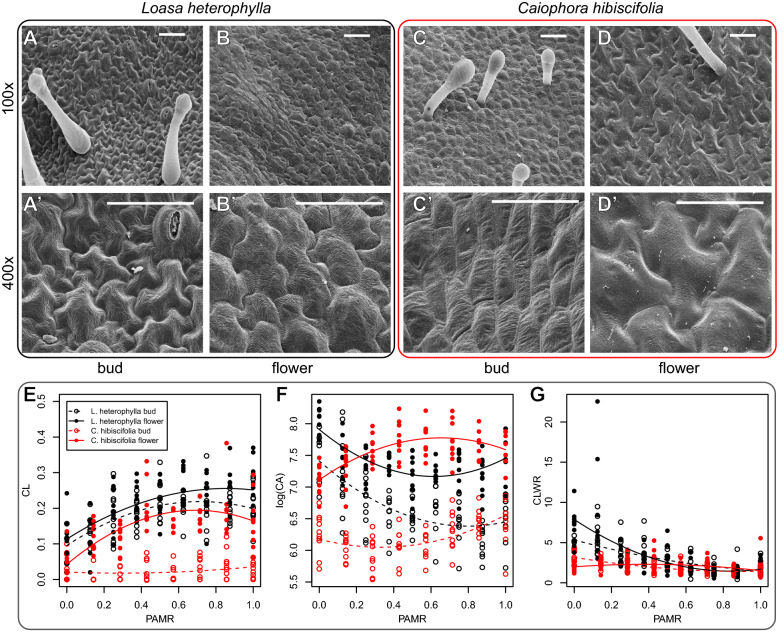
SEM images of the adaxial petal epidermis in 5 mm flower buds and mature flowers of *L. heterophylla* (**A**, **Aʹ**, **B**, **Bʹ**) and *C. hibiscifolia* (**C**, **Cʹ**, **D**, **Dʹ**). Relationship between cell lobeyness (CL) and position of SEM image along the petal midrib (PAMR) (**E**); relationship between the logarithm of cell area, *log*(CA), and PAMR (**F**), and relationship between cell length-to-width ratio (CLWR) and PAMR (**G**) in 5 mm flower buds and mature flowers of *L. heterophylla* and *C. hibiscifolia*. PAMR goes from basal (0), to apical (1)

Two regressions were used to test predictions about cell morphology, i.e., CL and *log*(CA), along the petal midrib in *L. heterophylla* and *C. hibiscifolia*, under a scenario of delayed differentiation of epidermal cells in the later (Fig. 2). The first regression included an interaction among PAMR, developmental stage and species affecting CL; the second regression an interaction among PAMR, developmental stage and species affecting *log*(CA). CL is higher towards the petal apex in flower buds of *L. heterophylla* (*t* = 5.404, *P* < 0.0001; *t* = − 4.031, *P* < 0.0001, for the linear and the quadratic effects of PAMR, respectively) (Fig. 3e). CL tends to be higher in mature flowers of *L. heterophylla* when compared to its 5 mm buds (Fig. 3e), but this difference is not significant (*t* = 0.948, *P* = 0.344). The shape of the relationship between CL and PAMR does also not differ between flower buds and mature flowers in this species (*t* = − 0.159, *P* = 0.873 for the PAMR linear term × developmental stage interaction; *t* = 0.582, *P* = 0.561 for the PAMR quadratic term × developmental stage interaction) (Fig. 3e). CL is close to 0 along the whole petal midrib of 5 mm buds of *C. hibiscifolia* (*t* = − 4.118, *P* < 0.0001 for the PAMR linear term × species interaction; *t* = 3.285, *P* < 0.0001 for the PAMR quadratic term × species interaction) (Fig. 3e), but it increases towards the petal apex in mature flowers of this species (*t* = 3.806, *P* < 0.0005 for the PAMR linear term × developmental stage × species interaction; *t* = − 3.365, *P* < 0.001 for the PAMR quadratic term × developmental stage × species interaction) (Fig. 3e). *log*(CA) is reduced towards the petal apex in flower buds and mature flowers of *L. heterophylla* (*t* = − 6.450, *P* < 0.0001; *t* = − 10.150, *P* < 0.0001, for the linear and the quadratic effects of PAMR, respectively) (Fig. 3f). *log*(CA) is on average higher in mature flowers of *L. heterophylla* when compared to its 5 mm buds (*t* = 4.121, *P* < 0.0001), but the relationship between *log*(CA) and PAMR does not change in mature flowers of this species (*t* = 0.333, *P* = 0.739 for the PAMR linear term × developmental stage interaction; *t* = 0.626, *P* = 0.532 for the PAMR quadratic term × developmental stage interaction) (Fig. 3f). *log*(CA) increases moderately towards the petal apex along the midrib of 5 mm buds of *C. hibiscifolia* (*t* = 3.279, *P* < 0.005 for the PAMR linear term × species interaction; *t* = − 0.916, *P* = 0.360 for the PAMR quadratic term × species interaction) (Fig. 3f). *log*(CA) is on average higher in mature flowers of this species when compared to its flower buds (*t* = 2.623, *P* < 0.05 for the developmental stage × species interaction). *log*(CA) also increases moderately towards the apex along the midrib of mature flowers in *C. hibiscifolia*, but the curvature of the relationship between *log*(CA) and PAMR becomes inverted in mature flowers (*t* = 3.347, *P* < 0.001 for the PAMR linear term × developmental stage × species interaction; *t* = –4.048, *P* = 0.0001 for the PAMR quadratic term × developmental stage × species interaction) (Fig. 3f). In short, cell wall lobeyness is basipetal in mature flowers of the two species, but this pattern emerges later during flower development in *C. hibiscifolia*. *log*(CA) increases during flower development in both *L. heterophylla* and *C. hibiscifolia*, but the increase is more pronounced in the later. The shape of the relationship between *log*(CA) and PAMR does not change during the development of *L. heterophylla* but it does changes during the development of *C. hibiscifolia*. *log*(CA) does not differ remarkably between mature flowers of *L. heterophylla* and *C. hibiscifolia*.

A third and last regression was used to test if differences in corolla opening between mature flowers of *L. heterophylla* and *C. hibiscifolia* can be attributed to differences in cell elongation during petal development. This regression included an interaction among PAMR, developmental stage and species affecting CLWR. CLWR is higher at the petal base of 5 mm flower buds in *L. heterophylla* (*t* = − 3.087, *P* < 0.005; *t* = 1.287, *P* = 0.199, for the linear and the quadratic effects of PAMR, respectively) (Fig. 3g). CLWR increases disproportionally at the petal base in mature flowers of this species (*t* = − 3.269, *P* < 0.005 for the linear effect of PAMR × developmental stage interaction PAMR; *t* = 2.542, *P* < 0.05 for the quadratic effect of PAMR × developmental stage interaction) (Fig. 3g). CLWR is more reduced at the petal base of *C. hibiscifolia* buds when compared to *L. heterophylla* buds (*t* = − 3.513, *P* < 0.001). However, the pattern of CLWR along the petal midrib does not differ between buds of *L. heterophylla* and buds of *C. hibiscifolia* (*t* = 0.860, *P* = 0.390 for the linear effect of PAMR × species interaction PAMR; *t* = − 0.135, *P* = 0.892 for the quadratic effect of PAMR × species interaction) (Fig. 3g). CLWR at the petal base decreases in mature flowers of *C. hibiscifolia* (*t* = 3.685, *P* < 0.0005 for the linear effect of PAMR × developmental stage × species interaction PAMR; *t* = − 2.880, *P* < 0.005 for the quadratic effect of PAMR × developmental stage × species interaction) (Fig. 3g). Summing up, cell elongation takes place during petal development in *L. heterophylla* and does not take place during petal development in *C. hibiscifolia*. Indeed, this species seems to undergo cell widening at the petal base.

Epidermal cell number estimation for petals in the 5 mm flower buds and in mature flowers of *L. heterophylla* yielded 31,452 ± 3647.86 (SE) and 50,188 ± 4047.90 (SE) cells, respectively; epidermal cell number estimation for the 5 mm flower bud and the mature flower in *C. hibiscifolia* yielded 65,410 ± 4104.48 (SE) and 68,973 ± 4707.98 (SE) cells, respectively. Hence, cell number in 5 mm flower buds of *C. hibiscifolia* is higher than in 5 mm flower buds of *L. heterophylla*. Cell number increases during late flower development in *L. heterophylla* but does not increase during late flower development in *C. hibiscifolia*.

### DGE analysis

Samples cluster according to our sampling design and we did not find outliers (Additional file 2). We, therefore, included all samples in the subsequent differential gene expression analyses. We found 7465 DEGs between buds of *L. heterophylla* and buds of *C. hibiscifolia* (amounting to almost half of the total annotated transcripts) and 9003 DEGs between mature flowers of each species. For intraspecific contrasts, we found 3737 DEGs between flower buds and mature flowers of *L. heterophylla*, and 5304 DEGs between flower buds and mature flowers of *C. hibiscifolia*. DEGs found between flower buds and mature flowers that are common in their expression trend when the two species are compared can be consulted in Additional file 2.

### Gene set enrichment analysis

**Fig. 4:**
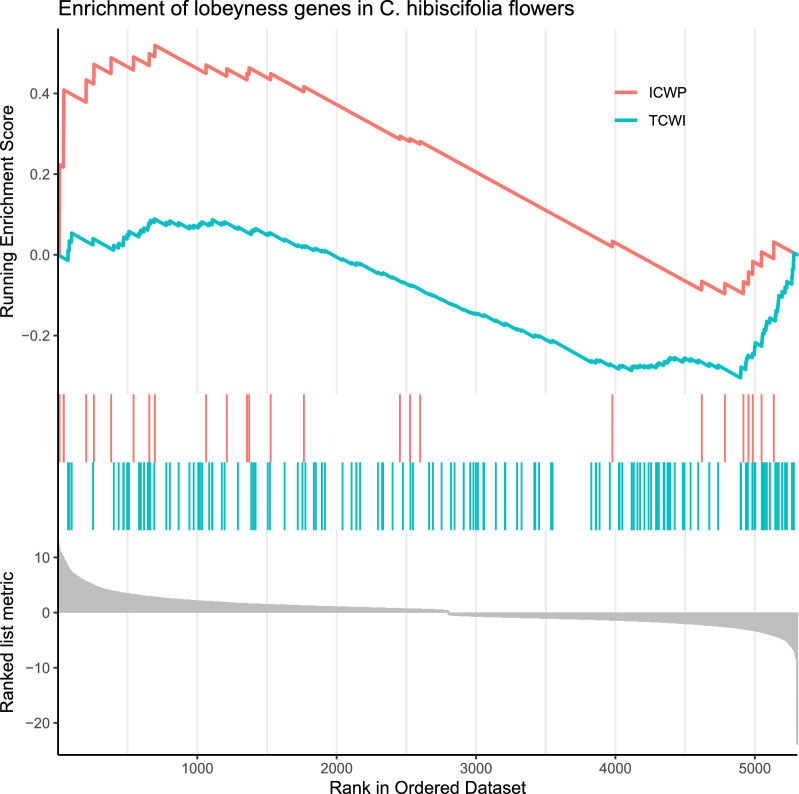
Enrichment of two signatures of lobeyness genes in *C. hibiscifolia*: *TCWI* turgor pressure-cell wall interaction genes, *ICWP* intrinsic cell wall properties genes. The lower values along the ‘Rank in Ordered in Dataset’ correspond to genes that are highly expressed in mature flowers; the higher values along this axis correspond to genes that are highly expressed in flower buds, based on *log2*(fold-change)

TCWI (turgor pressure-cell wall interaction) and ICWP (intrinsic cell wall property) signatures, where significantly enriched in the *C. hibiscifolia* 5 mm bud vs*. C. hibiscifolia* mature flower contrast (enrichment score = − 0.305, *P*-adjusted value < 0.001; enrichment score = 0.519, *P*-adjusted value < 0.05). The two signatures are enriched in genes that are either highly expressed in mature flowers or in 5 mm flower buds (Fig. 4). None of these signatures were enriched in the three remaining contrasts. The CE (cell elongation) and the FDM (flower differentiation–maturation) signatures were not enriched in any of the four contrasts.

## Discussion

We investigated whether the evolution of a large pedomorphic corolla in the hummingbird-pollinated *C. hibiscifolia* species resulted from delayed epidermal cell wall differentiation (Fig. 2). Under this scenario, we expected delayed development of basipetal cell wall lobeyness in *C. hibiscifolia* and increased cell growth during late petal development in this species when compared to the non-pedomorphic, *L. heterophylla* species. We also expected enrichment of gene sets related to cell wall lobeyness when comparing mature flowers to pre-anthesis buds in *C. hibiscifolia*, and no enrichment of these gene sets when comparing mature flowers to pre-anthesis buds in *L. heterophylla*. We found that mature flowers of *L. heterophylla* and *C. hibiscifolia* share the same basipetal cell wall lobeyness pattern along the petal midrib, but basipetal cell wall lobeyness develops later in *C. hibiscifolia* (Fig. 3e). In agreement with our hypothesis, overall increase in cell area is more pronounced when comparing 5 mm flower buds to mature flowers in *C. hibiscifolia* (Fig. 3f). Moreover, the GSEA of the contrast of 5 mm buds vs. mature flowers in *C. hibiscifolia* showed that the transcriptional profile of buds was enriched in gene sets that turn regular cell walls into the lobed cell walls (Fig. 2). Enrichment of these gene sets was not detected in the contrast of 5 mm buds vs. mature flowers in *L. heterophylla*. Thus, the GSEA of lobeyness genes suggest that cell wall lobeyness in Loasoideae is related to molecular processes that involve cell wall turgor pressure interactions, as well molecular processes that are intrinsic to the cell wall.

Cell geometry data and transcription profiles in pre-anthesis flower buds and mature flowers of the two analysed species suggest that delayed epidermal cell wall differentiation underlie to a large extent the evolution of large pedomorphic corollas in *C. hibiscifolia*. Nevertheless, we must be cautious about this cause-and-effect inference, as delayed lobeyness of epidermal cells may not be the cause of pedomorphosis in *C. hibiscifolia*, but a consequence of petal growth ceasing later in this species for other reasons. Caution must also be taken when interpreting the degree of petal pedomorphosis during the evolution of hummingbird pollination in *C. hibiscifolia*. When compared to bee-pollinated *Caiophora* species, *L. heterophylla* presents highly reflexed petals and remarkably open corollas. Corolla morphology may, therefore, be derived and peramorphic in *L. heterophylla*, rather than representing the morphology of the bee-pollinated ancestor of *C. hibiscifolia*. If this were the case, *C. hibiscifolia* would seem more pedomorphic than it really is. A final caveat in this study relates to the fact that *L. heterophylla* and *C. hibiscifolia* belong to different Loasoideae genera and diverged from a common ancestor around 40 Ma. [6]. Since inferential power decreases rapidly with phylogenetic distance, it would be ideal to repeat this comparison of cell geometry and transcription profiles between bee- and hummingbird-pollinated *Caiophora* species, which diverged from a common ancestor less than 10 Ma. [6].

At the cellular level, heterochronic variation in plants has been mainly related to changes in the timing of cell proliferation and cell expansion [5]. Our study suggests that cell differentiation in tissues that are key in determining the growth dynamics of plant organs can also contribute to the generation of heterochronic variation in plants. Moreover, cell wall lobeyness is underpinned by molecular mechanisms that are shared between monocots and eudicots [42], and basipetal cell wall lobeyness characterizes leaf differentiation in other flowering plant lineages [18]. Hence, basic and highly conserved developmental mechanisms that underlie cell wall lobeyness in leaves may have been recruited for the generation of heterochronic variation in plant lineages that present jigsaw puzzle-shaped cells on their differentiated petal epidermis, e.g., *Aquilegia* (Ranunculaceae), *Centranthus* (Valerianaceae) [24, 28] and several species of the Spiraeeae tribe (Rosaceae) [32]. This is an interesting finding, as most studies exploring the molecular basis of variation in flower morphology search for differential expression in “flower” genes, which are usually transcription factors, such as CYC or MADS-box genes [12].

Developmental repatterning in general involves not a single, but an array of developmental mechanisms [1]. In our case, increased cell proliferation at early developmental stages, a form of heterometry [1] and arrest of cell elongation at the petal base during late development in *C. hibiscifolia* (Fig. 3g), a form of developmental suppression, may have contributed, together with pedomorphosis, to the corolla morphology of hummingbird-pollinated flowers. Cell number in *C. hibiscifolia* 5 mm flower buds is higher than in 5 mm buds of *L. heterophylla*. Cell number does not increase remarkably when comparing the 5 mm bud of *C. hibiscifolia* to its mature flower. Hence, cell proliferation before the 5 mm flower bud stage may have contributed to large corolla size in *C. hibiscifolia*. Moreover, disproportionate cell growth at the petal base accounts only partially for the expanded petal base of *L. heterophylla*. Regular cells at the petal base in *L. heterophylla* are comparatively larger than the jigsaw puzzle-shaped cells towards the petal apex (Fig. 3e, f), which suggests that cell expansion at the petal base took place in previous developmental stages. However, cell area does not increase proportionally more, at the petal base, during flower anthesis in this species (Fig. 3f). Interspecific differences in petal base expansion at flower maturity can instead be attributed to disproportionate cell elongation at the petal base in *L. heterophylla*, and arrest of cell elongation in *C. hibiscifolia* (Fig. 3g).

Interestingly, not only petals, but also the nectar containers, which are important to the plant–pollinator interaction in Loasoideae [2], are pedomorphic in *Caiophora* hummingbird-pollinated species [35, 37]. To what extent the cellular and molecular mechanisms described in this work affect not only the petals but also other flower whorls is a matter that deserves further exploration. Moreover, further exploration at single-cell resolution during flower development might add to the understanding of the cellular and morphogenetic patterns reported in this study.

## Conclusion

Our study highlights the complex nature of developmental repatterning and evolutionary change in flower morphology. Pedomorphosis, along with other mechanisms of developmental repatterning, likely resulted in the morphology of hummingbird-pollinated Loasoideae flowers. Our results also suggest that variation in flower morphology may originate in developmental processes that are shared between flower organs and leaves, at least in plant lineages that present jigsaw puzzle-shaped cells on their petal epidermis. Furthermore, our findings highlight that alternative approaches to a flower-centric perspective can yield useful insights to our understanding of the developmental basis of corolla morphology.

## Supplementary Information

**Additional file 1.** Phylogeny of Loasoideae including reconstructions of the ancestral pollinator and the ancestral flower morphology.

**Additional file 2.** R scripts used in RNAseq data analyses and in cell geometry data analyses. Html version.

**Additional file 3.** Cell geometry data.

**Additional file 4.** Assembly stats.

**Additional file 5.** *Camptotheca acuminata* gene models.

**Additional file 6.** *Caiophora hibiscifolia* isoform counts.

**Additional file 7.** *Caiophora hibiscifolia* blastx vs. *Camptotheca acuminata* peptides.

**Additional file 8.** *Camptotheca acuminata* gene to transcript mapping.

**Additional file 9.** *Camptotheca acuminata* Trinotate annotation report.

**Additional file 10.** *Loasa heterophylla* isoform counts.

**Additional file 11.** *Loasa heterophylla* blastx vs. *Camptotheca acuminata* peptides.

**Additional file 12.** R scripts used in RNAseq data analyses and in cell geometry data analyses. Rmd version.

**Additional file 13.** GO enrichment results for the *Loasa heterophylla* flower bud vs. *Caiophora hibiscifolia* flower bud contrast. Significance level was set to 0.05.

**Additional file 14.** GO enrichment results for the *Caiophora hibiscifolia* flower bud vs. *Caiophora hibiscifolia* mature flower contrast. Significance level was set to 0.05.

**Additional file 15.** GO enrichment results for the *Loasa heterophylla* mature flower vs. *Caiophora hibiscifolia* mature flower contrast. Significance level was set to 0.05.

**Additional file 16.** GO enrichment results for the *Loasa heterophylla* flower bud vs. *Loasa heterophylla* mature flower contrast. Significance level was set to 0.05.

**Additional file 17.** GO enrichment results for the *Loasa heterophylla* flower bud vs. *Loasa heterophylla* mature flower contrast. Wilcox. Log2FoldChange.

**Additional file 18.** GO enrichment results for the *Loasa heterophylla* flower bud vs. *Loasa heterophylla* mature flower contrast. Wilcox. P-value.

**Additional file 19.** *Camptotheca acuminata* gene models. Functional annotation.

**Additional file 20.** RNAseq sample information.

## Data Availability

R scripts used in the cell morphology and transcriptome analyses were uploaded as Additional file 2. Cell morphology data supporting the results reported in this article has also been uploaded in Additional file 3. Data generated and used during transcriptome analysis were uploaded as Additional files 4, 5, 6, 7, 8, 9, 10, 11, 12, 13, 14, 15, 16, 17, 18, 19 and 20. Raw sequencing files are available through NCBI’s Short Read Archive (BioProject accession: PRJNA763894).
