# Correction to: Delayed differentiation of epidermal cells walls can underlie pedomorphosis in plants: the case of pedomorphic petals in the hummingbird-pollinated *Caiophora hibiscifolia* (Loasaceae, subfam. Loasoideae) species

**DOI:** 10.1186/s13227-022-00193-6

**Published:** 2022-02-15

**Authors:** Marina M. Strelin, Eduardo E. Zattara, Kristian K. Ullrich, Mareike Schallenberg‑Rüdinger, Stefan A. Rensing

**Affiliations:** 1 Grupo de Investigación en Ecología de La Polinización, Laboratorio Ecotono, INIBIOMA (CONICET - Universidad Nacional del Comahue), San Carlos de Bariloche, Río Negro, Argentina; 2 Department of Evolutionary Biology, August Thienemann Str. 2, 24306 Plön, Germany; 3 IZMB - Institut Für Zelluläre Und Molekulare Botanik, Abt. Molekulare Evolution, University of Bonn, Kirschallee 1, 53115 Bonn, Germany; 4 Plant Cell Biology, Department of Biology, University of Marburg, Marburg, Germany

## Correction to: Strelin et al. EvoDevo (2022) 13:1 https://doi.org/10.1186/s13227-021–00186-x

In this article [1], the mid initials of two of the co-authors were missing. It has been added in this correction.

Stefan (A.) Rensing and Kristian (K.) Ullrich are the full names of the co-authors.
