# Supplementary material for: Delayed differentiation of epidermal cells walls can underlie pedomorphosis in plants: the case of pedomorphic petals in the hummingbird-pollinated Caiophora hibiscifolia (Loasaceae, subfam. Loasoideae) species
Source: EvoDevo. 2022 Jan 3;13:1. doi: 10.1186/s13227-021-00186-x (PMC8725396; doi:10.1186/s13227-021-00186-x)
Supplement: Supplementary file 1 — Additional file 1. Phylogeny of Loasoideae including reconstructions of the ancestral pollinator and the ancestral flower morphology. [file 13227_2021_186_MOESM1_ESM.pdf]

Supplementary material S1. Stochastic character mappings of a, b the pollinator and c, d the flower phenotype. Pollinationc strategies and flower phenotype are indicated with colours. Pollination strategy: blue = bee pollination; green = hummingbird pollination; purple = rodent pollination; flower phenotype: red = tilt-revolver flower; cyan = funnel-revolver flower. Panels A and C show the maximum credibility tree with pie charts on its nodes, indicating the posterior probability of a each pollination strategy and c each flower phenotype retrieved by 1000 stochastic character mappings. Panels B and D show the superimposition of 1000 phylogenetic trees, with 10 stochastic character mappings each. The species in this study are shown with arrows. Elements of this figure were taken from Strelin et al. 2016.

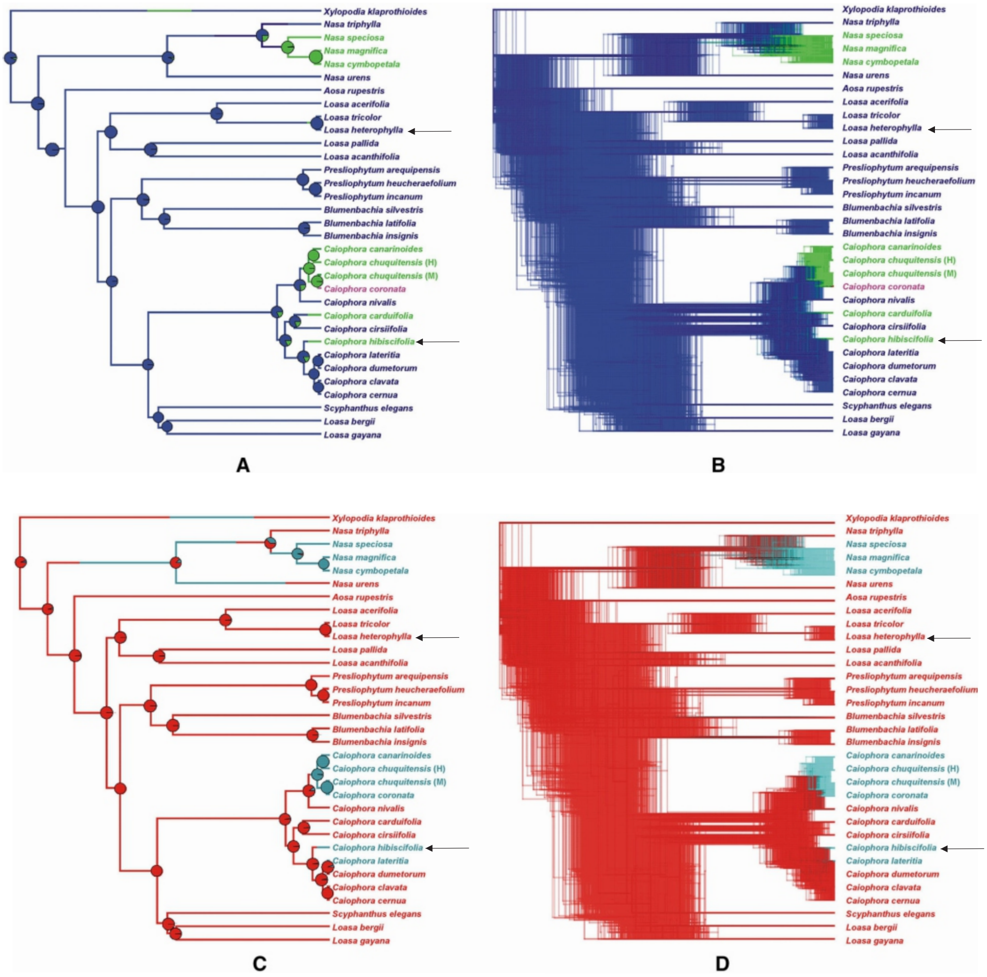

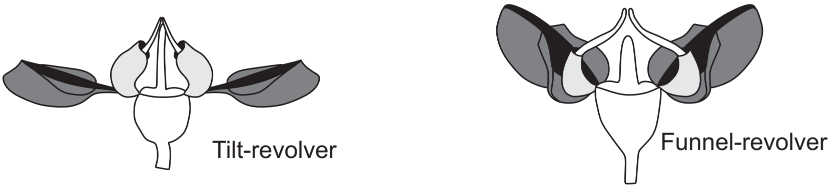

REFERENCES

Strelin MM, Benitez-Vieyra S, Ackermann M, Cocucci AA. Flower reshaping in the transition to hummingbird pollination in Loasaceae subfam. Loasoideae despite absence of corolla tubes or spurs. Evol. Ecol. 2016a; 30(3): 401-417.
