# Supplementary material for: Delayed differentiation of epidermal cells walls can underlie pedomorphosis in plants: the case of pedomorphic petals in the hummingbird-pollinated Caiophora hibiscifolia (Loasaceae, subfam. Loasoideae) species
Source: EvoDevo. 2022 Jan 3;13:1. doi: 10.1186/s13227-021-00186-x (PMC8725396; doi:10.1186/s13227-021-00186-x)
Supplement: Supplementary file 2 — Additional file 2. R scripts used in RNAseq data analyses and in cell geometry data analyses. Html version. [file 13227_2021_186_MOESM2_ESM.html]

Evolution of pedomorphic petals in C. hibiscifolia


# Evolution of pedomorphic petals in C. hibiscifolia

### Supplementary Code and Results

#### Eduardo E. Zattara and Marina M. Strelin

#### 9/21/2020

## Library preparation and sequencing

RNA was extracted from petals of *Loasa heterophylla* (LOA) and *Caiophora hibiscifolia* (CAI) at two developmental stages: bud and flower (flo). 12 libraries were initially prepared, comprising 2 species x 2 stages x 3 biological replicates, and sequenced as 100 bp, single-end reads in the Illumina platform. After QC it was found that one of the LOA-bud libraries failed to be properly sequenced; preliminary data analysis evidenced that another of the LOA-bud libraries had been derived from a mislabeled sample, and corresponded to LOA flower tissue. This second sample was assigned to the LOA-flo group, and two additional libraries were prepared from frozen LOA bud tissues and sequenced as 150 bp, paired-end reads in the Illumina platform. All reads were stored as FastQ files.

## Initial Transcriptome Assembly

FastQ files for each species were quality trimmed with Trimmomatic, and fed to Trinity-v2.4.0 to assemble species-specific reference transcriptomes. Completeness of each assembly was assessed using BUSCO v4.1.3 with the embryophyta\_odb10 lineage dataset.

## *Camptotheca acuminata* genome

Since there is no available genome from any species within the family Loasaceae, available genomes from the closest possible families were considered as a source for annotated “common” reference. The genome from the Chinese Happy Tree, *Camptotheca acuminata* (**CAMac**, Family Nyssaceae) was chosen for this purpose https://doi.org./10.1093/gigascience/gix065. The gene models were annotated using Trinotate.

Fasta files containing transcript and peptide sequences for all gene models were retrieved from http://dx.doi.org/10.5061/dryad.nc8qr: `cac_hc_gene_models.cdna.fa`  
`cac_hc_gene_models.pep.fa`

A protein database for blast searches was made using makeblastd (requires NCBI’s Blast+ from ftp://ftp.ncbi.nlm.nih.gov/blast/executables/blast+/LATEST/)

```
     makeblastdb -in cac_hc_gene_models.pep.fa -dbtype prot
```

### Trinotate annotation of CAMac gene models

Although DataDryad’s files include a functional annotation table for CAMac gene models, this annotation only includes putative gene names. To generate additional annotations linked to Gene Ontology(GO) terms, Trinotate was used, in combination with Blast+, Transdecoder and SQLite. HHMER/PFAM and other tools used by Trinotate were not used.

Once installed, Trinotate’s boilerplate database, Uniprot/Swisprot and PFAM’s reference databases can be downloaded using the command

```
$TRINOTATE_HOME/admin/Build_Trinotate_Boilerplate_SQLite_db.pl ../Trinotate_Boilerplate/Trinotate
```

This will yield three files:

- `Trinotate.sqlite` Trinotate’s boilerplate database
- `uniprot_sprot.pep` Uniprot/Sprot reference database
- `Pfam-A.hmm.gz` Pfam’s database (not used here)

The protein database is indexed for blast searches using `makeblastd` (requires NCBI’s Blast+,

```
makeblastdb -in ../Trinotate_Boilerplate/uniprot_sprot.pep -dbtype prot
```

and then searched against using `cac_hc_gene_models.pep.fa` as query sequences.

```
blastp -query CAMac/cac_hc_gene_models.pep.fa \
   -db ../Trinotate_Boilerplate/uniprot_sprot.pep \
   -num_threads 6 -max_target_seqs 1 -outfmt 6 \
   -evalue 1e-3 > CAMac/CAMac.blastp_vs_uniprot_sprot.outfmt6 &
   
#Optional: Monitor output using tail
tail -f CAMac/CAMac.blastp_vs_uniprot_sprot.outfmt6
```

Trinotate needs peptide files with coordinates as given by the TransDecoder peptide prediction package

```
     $TRANSDECODER_HOME/TransDecoder.LongOrfs -t CAMac/cac_hc_gene_models.cdna.fa 
     $TRANSDECODER_HOME/TransDecoder.Predict -t CAMac/cac_hc_gene_models.cdna.fa
```

It also requires a gene-to-transcript table (since it is designed to deal with Trinity output). In this case, a list of all CAMac gene models repeated in two columns will serve.

First, we use bash `grep` to extract a list of gene names from the fasta file.

```
     grep '>' CAMac/cac_hc_gene_models.pep.fa | sed 's/>//g' > CAMac/cac_hc_gene_models.pep.names
```

That file is read in R, columns are duplicated and then exported.

```
#   Read list of gene names and duplicate as columns to generate the gene-to-transcript map needed for Trinotate
CAMac_names <- readr::read_table("cac_hc_gene_models.pep.names", col_names = FALSE)
```

```
## 
## -- Column specification --------------------------------------------------------
## cols(
##   X1 = col_character()
## )
```

```
CAMac_names %>% mutate(X2 = X1) -> CAMac_names
write_tsv(CAMac_names, file = "CAMac_gene_to_transcript.map", col_names = F)
```

Now everything necessary to fill the Trinotate database is ready.

```
#   Initialize Trinotate's database and load Camptotheca gene models
$TRINOTATE_HOME/Trinotate ../Trinotate_Boilerplate/Trinotate.sqlite init \
            --gene_trans_map CAMac/CAMac_gene_to_transcript.map \
            --transcript_fasta CAMac/cac_hc_gene_models.cdna.fa \
            --transdecoder_pep CAMac/cac_hc_gene_models.cdna.fa.transdecoder.pep

#   Load uniprot blast hits
$TRINOTATE_HOME/Trinotate ../Trinotate_Boilerplate/Trinotate.sqlite \
            LOAD_swissprot_blastp CAMac/CAMac.blastp_vs_uniprot_sprot.outfmt6

#   Export Trinotate's annotation report
     $TRINOTATE_HOME/Trinotate ../Trinotate_Boilerplate/Trinotate.sqlite \
            report > CAMac/CAMac_trinotate_annotation_report.tsv
```

Now that we have the Trinotate annotation report, we can import it to R and merge it with the existing annotations.

```
#Read annotations from Camptotheca annotation table
CAMac_trinotate_annot <- read_tsv("CAMac_trinotate_annotation_report.tsv", na = ".",) %>%
  select(geneID,transcript_id,sprot_Top_BLASTX_hit,ARAth_BLASTX, Kegg, gene_ontology_BLASTX)
```

```
## Rows: 41306 Columns: 19
```

```
## -- Column specification --------------------------------------------------------
## Delimiter: "\t"
## chr (10): geneID, transcript_id, sprot_Top_BLASTX_hit, prot_id, prot_coords,...
## lgl  (9): RNAMMER, sprot_Top_BLASTP_hit, Pfam, SignalP, TmHMM, gene_ontology...
```

```
## 
## i Use `spec()` to retrieve the full column specification for this data.
## i Specify the column types or set `show_col_types = FALSE` to quiet this message.
```

```
#and remove duplicate entries
CAMac_trinotate_annot <- CAMac_trinotate_annot[!duplicated(CAMac_trinotate_annot),]

#Include functional annotation table from http://dx.doi.org/10.5061/dryad.nc8qr
CAMac_annot <- read_delim("cac_hc_gene_models.func_anno.txt", delim = "\t", col_names=FALSE)
```

```
## Rows: 40332 Columns: 2
```

```
## -- Column specification --------------------------------------------------------
## Delimiter: "\t"
## chr (2): X1, X2
```

```
## 
## i Use `spec()` to retrieve the full column specification for this data.
## i Specify the column types or set `show_col_types = FALSE` to quiet this message.
```

```
names(CAMac_annot)<-c("geneID","annotation")

#Merge both tables
CAMac_annot <- left_join(CAMac_annot, CAMac_trinotate_annot) %>% select(-transcript_id)
```

```
## Joining, by = "geneID"
```

## Mapping *Loasa*’s and *Caiophora*’s transcripts to CAMac gene models and collapsing reads.

### Map *Loasa* counts to *Camptotheca* blast hits

Perform a blast search of all Trinity transcripts from *Loasa heterophylla* against *Camptotheca*’s peptides

```
blastx -query LOAhet/LOAhet.v3.Trinity.fasta \
       -db CAMac/cac_hc_gene_models.pep.fa \
       -num_threads 6 -max_target_seqs 1 -outfmt 6 \
       -evalue 1e-3 > LOAhet/LOAhet.v3.blastx_vs_CAMac_peptides.outfmt6 &

#Optional: Monitor output using tail
tail -f LOAhet/LOAhet.v3.blastx_vs_CAMac_peptides.outfmt6
```

Once the blast search is complete, results are imported.

```
#Import blast output
blast_outfmt6_headers <- c("query_id","CAMac_id","pident","length", "mismatch", "gapopen",                                                                "qstart","qend","sstart","send","evalue","bitscore")

loa_to_cam <- read_tsv("LOAhet.v3.blastx_vs_CAMac_peptides.outfmt6", 
               col_names = blast_outfmt6_headers)
```

```
## Rows: 78899 Columns: 12
```

```
## -- Column specification --------------------------------------------------------
## Delimiter: "\t"
## chr  (2): query_id, CAMac_id
## dbl (10): pident, length, mismatch, gapopen, qstart, qend, sstart, send, eva...
```

```
## 
## i Use `spec()` to retrieve the full column specification for this data.
## i Specify the column types or set `show_col_types = FALSE` to quiet this message.
```

```
loa_to_cam$geneID <- str_replace(loa_to_cam$query_id, "TRINITY", "LOAhetv3")
```

Then the RSEM count matrices at the isoform level are imported, and the `loa_to_cam` table is used to assign a CAMac gene model to *Loasa*’s isoforms that had a blast hit. Since an `inner_join` operation is used, all isoforms without a blast hit are discarded. Then, reads for all isoforms matching a single CAMac gene are collapsed by summing.

```
#Read count tables
loa_isoform_cts <- read.delim("LOAhet.RSEM.isoform.counts.matrix", row.names = 1) %>%
  as_tibble(rownames = "query_id") 

#Check that total fragment counts per sample are mostly similar
colSums(loa_isoform_cts[,2:8], na.rm = T)
```

```
## l_bud_rep1 l_bud_rep2 l_bud_rep3 l_flo_rep1 l_flo_rep2 l_flo_rep3 l_flo_rep4 
##   13336138   11615160   10490181   14128847   14757293   13340372   13838282
```

```
# Inner join to generate a CAI isoform to CAM transcript
loa_isof_cts_cam <- loa_to_cam %>% 
  select(query_id, CAMac_id) %>%
  inner_join(loa_isoform_cts)
```

```
## Joining, by = "query_id"
```

```
# Replace the word "TRINITY" to a species-specific term, useful if transcript names from both species are merged
loa_isof_cts_cam$query_id <- str_replace(loa_isof_cts_cam$query_id, "TRINITY", "LOAhetv3")

# Collapse all counts from isoforms mapping to the same Camptotheca gene
loa_isof_cts_cam %>% 
  select(-query_id) %>%
  group_by(CAMac_id) %>% 
  summarise_all(list(sum = sum)) -> loa_gene_cts_cam
loa_gene_cts_cam
```

```
## # A tibble: 18,594 x 8
##    CAMac_id       l_bud_rep1_sum l_bud_rep2_sum l_bud_rep3_sum l_flo_rep1_sum
##    <chr>                   <dbl>          <dbl>          <dbl>          <dbl>
##  1 Cac_g000002.t1        1090.           1260           1014          1262.  
##  2 Cac_g000004.t1          90.8           113            144.           93.9 
##  3 Cac_g000005.t1           2.75            8              0             4.51
##  4 Cac_g000007.t1         173              11             61            34   
##  5 Cac_g000010.t1        1716             830            684          1340   
##  6 Cac_g000012.t1          26.8            18.8           64.6          47.1 
##  7 Cac_g000014.t2         783.           2389.          1740.         2091.  
##  8 Cac_g000016.t2         132             129            127           138   
##  9 Cac_g000017.t1        1253.           1635.          1601.         4192.  
## 10 Cac_g000019.t1         125             152            107            74   
## # ... with 18,584 more rows, and 3 more variables: l_flo_rep2_sum <dbl>,
## #   l_flo_rep3_sum <dbl>, l_flo_rep4_sum <dbl>
```

### Map *Caiophora* counts to *Camptotheca* blast hits

Perform a blast search of all Trinity transcripts from *Caiophora hibiscifolia* against *Camptotheca*’s peptides

```
blastx -query CAIhib.v2.Trinity.fasta -db cac_hc_gene_models.pep.fa -num_threads 6 -max_target_seqs 1 -outfmt 6 -evalue 1e-3 > CAIhib.v2.blastx_vs_CAMac_peptides.outfmt6 &

blastx -query CAIhib/CAIhib.v2.Trinity.fasta \
       -db CAMac/cac_hc_gene_models.pep.fa \
       -num_threads 6 -max_target_seqs 1 -outfmt 6 \
       -evalue 1e-3 > CAIhib/CAIhib.v2.blastx_vs_CAMac_peptides.outfmt6 &

#Optional: Monitor output using tail
tail -f CAIhib/CAIhib.v2.blastx_vs_CAMac_peptides.outfmt6
```

Once the blast search is complete, results are imported.

```
#Import blast output
cai_to_cam <- read_tsv("CAIhib.v2.blastx_vs_CAMac_peptides.outfmt6", 
                       col_names = blast_outfmt6_headers)
```

```
## Rows: 83675 Columns: 12
```

```
## -- Column specification --------------------------------------------------------
## Delimiter: "\t"
## chr  (2): query_id, CAMac_id
## dbl (10): pident, length, mismatch, gapopen, qstart, qend, sstart, send, eva...
```

```
## 
## i Use `spec()` to retrieve the full column specification for this data.
## i Specify the column types or set `show_col_types = FALSE` to quiet this message.
```

Then the RSEM count matrices at the isoform level are imported, and the `cai_to_cam` table is used to assign a CAMac gene model to *Caiophora*’s isoforms that had a blast hit. Since an `inner_join` operation is used, all isoforms without a blast hit are discarded. Then, reads for all isoforms matching a single CAMac gene are collapsed by summing.

```
#Read count tables
cai_isoform_cts <- read.delim("CAIhib.RSEM.isoforms.counts.matrix", row.names = 1) %>%
  as_tibble(rownames = "query_id") 
#Check that total fragment counts per sample are mostly similar
colSums(cai_isoform_cts[,2:7], na.rm = T)
```

```
## c_bud_rep01 c_bud_rep02 c_bud_rep03 c_flo_rep01 c_flo_rep02 c_flo_rep03 
##    11762245    12727855    11044756    12135688    10165992    10938078
```

```
# Replace the word "TRINITY" to a species-specific term, useful if transcript names from both species are merged
cai_isoform_cts$query_id <- str_replace(cai_isoform_cts$query_id, "TRINITY", "CAIhibv2")

# Inner join to generate a CAI isoform to CAM transcript
cai_isof_cts_cam <- cai_to_cam %>% 
  select(query_id, CAMac_id) %>%
  inner_join(cai_isoform_cts)
```

```
## Joining, by = "query_id"
```

```
# Collapse all counts from isoforms mapping to the same Camptotheca gene
cai_isof_cts_cam %>% 
  select(-query_id) %>%
  group_by(CAMac_id) %>% 
  summarise_all(list(sum = sum)) -> cai_gene_cts_cam
```

### Merge *Loasa* and *Caiophora* reads based on *Camptotheca* gene matches

Sample (row) labels are simplified and both count tables are merged using an inner join so only collections of transcript counts with hits to CAMac in both species are retained.

```
sample.labels <- c("L.Bud.1","L.Bud.2","L.Bud.3","L.Flo.1","L.Flo.2","L.Flo.3","L.Flo.4","C.Bud.1","C.Bud.2", "C.Bud.3","C.Flo.1","C.Flo.2","C.Flo.3")
CAMac_loa_cai_cts <- inner_join(loa_gene_cts_cam, cai_gene_cts_cam)
```

```
## Joining, by = "CAMac_id"
```

```
names(CAMac_loa_cai_cts) <- c("geneID",sample.labels)
```

## Normalization and data exploration

### Normalizing using the trimmed mean of M-values

One simple yet robust way to estimate the ratio of RNA production uses a weighted trimmed mean of the log expression ratios (trimmed mean of M values (TMM) (Robinson & Oshlack 2010)). This normalizes by effective library size, but not feature length.

```
rnaseqMatrix <- CAMac_loa_cai_cts %>% select(-geneID) %>% as.matrix()
row.names(rnaseqMatrix) <-CAMac_loa_cai_cts$geneID
exp_study = edgeR::DGEList(counts=rnaseqMatrix, group=factor(colnames(rnaseqMatrix)))
exp_study = edgeR::calcNormFactors(exp_study)
exp_study$samples$eff.lib.size = exp_study$samples$lib.size * exp_study$samples$norm.factors
CAMac_loa_cai_TMM <- edgeR::cpm(rnaseqMatrix) %>% as_tibble(rownames = "geneID")
```

### Stats and data distribution

After generating a `DGElist` from the counts, the counts are normalized to counts-per-million (cpm) using the `edgeR::cpm` function. A filter is applied to remove any genes not having at least 1 cpm in at least three samples. Counts are then log2 transformed, and the table is pivoted to generate violin plots showing the distribution

```
myDGEList <- edgeR::DGEList(rnaseqMatrix)
# take a look at the DGEList object 
myDGEList
```

```
## An object of class "DGEList"
## $counts
##                L.Bud.1 L.Bud.2 L.Bud.3 L.Flo.1 L.Flo.2 L.Flo.3 L.Flo.4 C.Bud.1
## Cac_g000002.t1 1089.86 1260.00 1014.00 1262.11 1536.12 1415.57 1473.56  624.00
## Cac_g000005.t1    2.75    8.00    0.00    4.51   11.59    7.24    0.00   29.00
## Cac_g000007.t1  173.00   11.00   61.00   34.00   19.00   25.00   81.00  280.16
## Cac_g000010.t1 1716.00  830.00  684.00 1340.00 1176.00  674.00  768.00 1070.00
## Cac_g000012.t1   26.83   18.77   64.59   47.07   37.16   47.32   26.00    0.00
##                C.Bud.2 C.Bud.3 C.Flo.1 C.Flo.2 C.Flo.3
## Cac_g000002.t1  610.00  513.00    1078  876.00    1071
## Cac_g000005.t1   30.00   21.00      19   19.00      10
## Cac_g000007.t1  326.74  193.66     104  125.62      70
## Cac_g000010.t1 1030.00  947.00     634  560.00    1024
## Cac_g000012.t1    1.00    1.00       0    1.00       1
## 15549 more rows ...
## 
## $samples
##         group lib.size norm.factors
## L.Bud.1     1 12947254            1
## L.Bud.2     1 11356741            1
## L.Bud.3     1 10345445            1
## L.Flo.1     1 13868436            1
## L.Flo.2     1 14461767            1
## 8 more rows ...
```

```
#Get counts per million using the 'cpm' function from EdgeR
cpm <- edgeR::cpm(myDGEList) 
colSums(cpm)
```

```
## L.Bud.1 L.Bud.2 L.Bud.3 L.Flo.1 L.Flo.2 L.Flo.3 L.Flo.4 C.Bud.1 C.Bud.2 C.Bud.3 
##   1e+06   1e+06   1e+06   1e+06   1e+06   1e+06   1e+06   1e+06   1e+06   1e+06 
## C.Flo.1 C.Flo.2 C.Flo.3 
##   1e+06   1e+06   1e+06
```

```
log2.cpm <- cpm(myDGEList, log=TRUE)

# now set some cut-off to get rid of genes/transcripts with low counts
# again using rowSums to tally up the 'TRUE' results of a simple evaluation
# how many genes had more than 1e-7 CPM (TRUE) in all 13 samples
keepers <- rowSums(cpm>0.0000001)>=13 # Adjust this cutoff for the number of samples in the smallest group of comparisons.
# now use base R's simple subsetting method to filter your DGEList based on the logical produced above
myDGEList.filtered <- myDGEList[keepers,]
dim(myDGEList.filtered)
```

```
## [1] 12957    13
```

```
log2.cpm.filtered <- cpm(myDGEList.filtered, log=TRUE)
log2.cpm.filtered.df <- as_tibble(log2.cpm.filtered, rownames = "geneID")
colnames(log2.cpm.filtered.df) <- c("geneID", sample.labels)

# pivot this FILTERED data to make a tidyverse compatible table
log2.cpm.filtered.df.pivot <- pivot_longer(log2.cpm.filtered.df, # dataframe to be pivoted
                                           cols = 2:14, # column names to be stored as a SINGLE variable
                                           names_to = "samples", # name of that new variable (column)
                                           values_to = "expression") # name of new variable (column) storing all the values (data)
ggplot(log2.cpm.filtered.df.pivot) +
  aes(x=samples, y=expression, fill = samples) +
  geom_violin(trim = FALSE, show.legend = FALSE) +
  stat_summary(fun = "median", 
               geom = "point", 
               shape = 95, 
               size = 10, 
               color = "black", 
               show.legend = FALSE) +
  labs(y="log2 expression", x = "sample",
       title="Log2 Counts per Million (CPM)",
       subtitle="filtered, non-normalized",
       caption=paste0("produced on ", Sys.time())) +
  theme_bw()
```

### Principal component analysis (PCA)

After verifying that all samples have a similar distribution of expression values, a PCA is used to find which factors are driving the variance, test for potential batch effects, and examine overall distribution of samples in variable space.

```
#Read in design table
targets <- read_tsv("samples.txt") %>% mutate(group = paste0(species,"-",stage))
```

```
## Rows: 13 Columns: 6
```

```
## -- Column specification --------------------------------------------------------
## Delimiter: "\t"
## chr (5): sample, stage, species, seqtype, fastq
## dbl (1): batch
```

```
## 
## i Use `spec()` to retrieve the full column specification for this data.
## i Specify the column types or set `show_col_types = FALSE` to quiet this message.
```

```
#Identify variables of interest in study design file
group <- targets$group
group <- factor(group)

myDGEList.filtered.norm <- calcNormFactors(myDGEList.filtered, method = "TMM")

# use the 'cpm' function from EdgeR to get counts per million from your normalized data
log2.cpm.filtered.norm <- cpm(myDGEList.filtered.norm, log=TRUE)

pca.res <- prcomp(t(log2.cpm.filtered.norm), scale.=F, retx=T)

#look at the PCA result (pca.res) that you just created
summary(pca.res) # Prints variance summary for all principal components.
```

```
## Importance of components:
##                            PC1     PC2      PC3      PC4      PC5      PC6
## Standard deviation     98.4035 56.9264 37.21101 29.58580 25.97060 22.91046
## Proportion of Variance  0.5563  0.1862  0.07955  0.05029  0.03875  0.03016
## Cumulative Proportion   0.5563  0.7425  0.82206  0.87235  0.91110  0.94125
##                             PC7      PC8      PC9     PC10    PC11    PC12
## Standard deviation     18.08196 14.56551 13.49578 11.61583 10.3886 7.64351
## Proportion of Variance  0.01878  0.01219  0.01046  0.00775  0.0062 0.00336
## Cumulative Proportion   0.96004  0.97223  0.98269  0.99044  0.9966 1.00000
##                             PC13
## Standard deviation     1.298e-13
## Proportion of Variance 0.000e+00
## Cumulative Proportion  1.000e+00
```

```
screeplot(pca.res) # A screeplot is a standard way to view eigenvalues for each PCA
```

```
pc.var<-pca.res$sdev^2 # sdev^2 captures these eigenvalues from the PCA result
pc.per<-round(pc.var/sum(pc.var)*100, 1) # we can then use these eigenvalues to calculate the percentage variance explained by each PC
pc.per
```

```
##  [1] 55.6 18.6  8.0  5.0  3.9  3.0  1.9  1.2  1.0  0.8  0.6  0.3  0.0
```

Half of the variance is explained by PC1, and another 18% is explained by PC2. To explore the influence of each grouping variable in a series of barplots of the loading of each sample.

```
# Create a PCA 'small multiples' chart ----
# this is another way to view PCA loading to understand impact of each sample on each principal component
pca.res.df <- pca.res$x[,1:4] %>% 
  as_tibble() %>%
  add_column(sample = sample.labels,
             group = group,
             stage = targets$stage,
             batch = as.factor(targets$batch),
             seqtype = targets$seqtype,
             species = targets$species)

pca.pivot <- pivot_longer(pca.res.df, # dataframe to be pivoted
                          cols = PC1:PC4, # column names to be stored as a SINGLE variable
                          names_to = "PC", # name of that new variable (column)
                          values_to = "loadings") # name of new variable (column) storing all the values (data)

pspecies <- ggplot(pca.pivot) +
  aes(x=sample, y=loadings, fill=species) +
  geom_bar(stat="identity") +
  facet_wrap(~PC) +
  labs(title="Species",
       caption=paste0("produced on ", Sys.time())) +
  theme_bw() +
  coord_flip()

pstage <- ggplot(pca.pivot) +
  aes(x=sample, y=loadings, fill=stage) + 
  geom_bar(stat="identity") +
  facet_wrap(~PC) +
  labs(title="Stage",
       caption=paste0("produced on ", Sys.time())) +
  theme_bw() +
  coord_flip()

pbatch <- ggplot(pca.pivot) +
  aes(x=sample, y=loadings, fill=batch) + 
  geom_bar(stat="identity") +
  facet_wrap(~PC) +
  labs(title="Batch",
       caption=paste0("produced on ", Sys.time())) +
  theme_bw() +
  coord_flip()
```

PC1 shows that half of the variance is clearly driven by species.

```
pspecies
```

PC2, explaining 18.6% of the variance, responds to stage. The absolute value of the loadings are much larger for *Caiophora* samples.

```
pstage
```

Finally, we can see that none of the first four PCs (which together explain 87.2% of the variance) is solely driven by batch, although PC3 (8%) seems to show some batch effect.

```
pbatch
```

Thus, we can get an idea of how samples relate to each other by plotting them against the first two PCs, which cover 74.2% of the variance.

```
# Visualize your PCA result

pca.res.df <- as_tibble(pca.res$x)
ggplot(pca.res.df) +
  aes(x=PC1, y=PC2, label=sample.labels, color=group) +
  geom_point(size=4) +
  #  geom_label() +
#  stat_ellipse() +
  xlab(paste0("PC1 (",pc.per[1],"%",")")) + 
  ylab(paste0("PC2 (",pc.per[2],"%",")")) +
  labs(title="PCA plot",
       caption=paste0("produced on ", Sys.time())) +
  coord_fixed() +
  theme_bw()
```

As expected, samples from each species are widely separated by PC1, while PC2 separates stages. Interestingly the distance between stages is larger for *Caiophora* than for *Loasa*. Although there is a noticeable difference between the first **LOA-het-bud** sample and the next two (generated in a separate batch), they still point in the same direction; furthermore the latter are closer to the flower stage, thus reducing rather than biasing the stage-driven variance.

From this exploratory results, it will be expected that most differential gene expression will be found when comparing across species, with all possible comparisons yielding approximately the similarly sized DEG repertoires. In contrast, we would expect that contrasts between stages will yield a larger DGE repertoire in *Caiophora* than in *Loasa*.

## Differential gene expression analyses

Differentially expressed genes (DEGs) can be detected using several approaches. Here we use the DESeq2 approach, which estimates variance-mean dependence in count data and tests for DEGs using a model based on the negative bionomial distribution.

### Gather sample design information, build dds object and run DESeq

```
#Read sample information table
sampleColdata <- read.delim("samples.txt")
sampleColdata <- sampleColdata %>% mutate(sp_stage = paste(species,stage, sep = "_"))
sampleColdata$batch <- as.factor(sampleColdata$batch)

rownames(sampleColdata) <- sampleColdata$sample

gene_cts <- CAMac_loa_cai_cts %>% select(-geneID) %>% round()
gene_cts <- as.data.frame(gene_cts[,rownames(sampleColdata)])
rownames(gene_cts) <- CAMac_loa_cai_cts$geneID

#check all sample names are in column names, in the same order
all(rownames(sampleColdata) %in% colnames(gene_cts)) # Debe devolver "TRUE"
```

```
## [1] TRUE
```

```
all(rownames(sampleColdata) == colnames(gene_cts)) # Debe devolver "TRUE"
```

```
## [1] TRUE
```

```
#Assemble DESeq Data set object
dds_clc <- DESeqDataSetFromMatrix(countData = gene_cts,
                                  colData = sampleColdata,
                                  design = ~ sp_stage)
```

```
## converting counts to integer mode
```

```
#Run DESeq 
dds_clc <- DESeq(dds_clc)
```

```
## estimating size factors
```

```
## estimating dispersions
```

```
## gene-wise dispersion estimates
```

```
## mean-dispersion relationship
```

```
## final dispersion estimates
```

```
## fitting model and testing
```

### DEGs between stages in *Loasa*

Once the DESeq object has been created and analyzed, it is possible to use `results` to extract the results from specific contrasts. The false discovery rate (FDR) threshold for significance is set to 0.05.

```
# Extract the results of the specific contrasts 
res.LOA.flo_vs_bud <- results(dds_clc, contrast = c("sp_stage", "LOAhet_flo", "LOAhet_bud"), alpha=0.05)

summary(res.LOA.flo_vs_bud)
```

```
## 
## out of 15554 with nonzero total read count
## adjusted p-value < 0.05
## LFC > 0 (up)       : 1824, 12%
## LFC < 0 (down)     : 1913, 12%
## outliers [1]       : 182, 1.2%
## low counts [2]     : 0, 0%
## (mean count < 1)
## [1] see 'cooksCutoff' argument of ?results
## [2] see 'independentFiltering' argument of ?results
```

Almost one-quarter of the genes are DE between bud and flower stages in *Loasa*, distributed about equally in up- and downregulated genes.

An MA plot shows the log2 fold changes attributable to stage over the mean of normalized counts for all the samples. Colored points indicate DEGs.

```
# MA Plot
plotMA(res.LOA.flo_vs_bud)
```

Another way of visualizing the distribution is a volcano plot. The volcano plot shows up- (right) or down- (left) regulation on an inverted log scale so that genes showing a smaller adjusted *p-value* are higher. DEGs at the chosen level are shown in red.

```
#Volcano Plots
res.LOA.flo_vs_bud.tib <- as_tibble(res.LOA.flo_vs_bud, rownames = "geneID")

ggplot(res.LOA.flo_vs_bud.tib%>% filter(padj>=0.05)) +
  aes(y=-log10(padj), x=log2FoldChange) +
  geom_point(size=2, colour = "black", alpha=.2)+ 
  xlim (-10,10) + ylim(0,40)+ 
  geom_point(data=res.LOA.flo_vs_bud.tib %>% filter(padj<0.05), 
             colour = "red", alpha=.2) +
  labs(title="Loasa heterofila",
       subtitle = "Flower vs petal stage",
       caption=paste0("produced on ", Sys.time())) +
  theme_bw()
```

After defining the set of DEGs, they can be extracted and clustered to generate a heatmap of the *Loasa* samples.

```
# Subset DEGs (padj < 0.05)
sig.05.res.LOA.flo_vs_bud.tib <- res.LOA.flo_vs_bud.tib %>% filter(padj<0.05) %>% arrange(desc(padj))

# Cluster DEGs and generate a heatmap for Loasa samples
#Convert TMM expression values into matrix
sig.05.res.LOA.flo_vs_bud.TMM <- CAMac_loa_cai_TMM[,1:8] %>% 
  filter(geneID %in% sig.05.res.LOA.flo_vs_bud.tib$geneID) 
geneIDs <- sig.05.res.LOA.flo_vs_bud.TMM$geneID

sig.05.res.LOA.flo_vs_bud.TMM.matrix <- sig.05.res.LOA.flo_vs_bud.TMM %>% 
  select(-geneID) %>% 
  as.matrix()
rownames(sig.05.res.LOA.flo_vs_bud.TMM.matrix) <- sig.05.res.LOA.flo_vs_bud.TMM$geneID

#clustering  genes (rows) in each DEG set
# we use the 'cor' function and the Pearson method for finding all pairwise correlations of genes
# '1-cor' converts this to a 0-2 scale for each of these correlations, which can then be used to 
# calculate a distance matrix using 'as.dist'
clustRows <- hclust(as.dist(1-cor(t(sig.05.res.LOA.flo_vs_bud.TMM.matrix), method="pearson")), method="complete") 

#clustering samples (columns) by spearman correlation
clustColumns <- hclust(as.dist(1-cor(sig.05.res.LOA.flo_vs_bud.TMM.matrix, method="spearman")), method="complete") #cluster columns 
#note: we use Spearman, instead of Pearson, for clustering samples because it gives equal weight to highly vs lowly expressed transcripts or genes

#Cut the resulting tree in k=2 clusters. k migh be changed to better fit the data   
module.assign <- cutree(clustRows, k=2)

#create color vector for clusters 
module.color <- rainbow(length(unique(module.assign)), start=0.1, end=0.9) 
module.color <- module.color[as.vector(module.assign)] 
myheatcolors2 <- colorRampPalette(colors=c("blue","white","red"))(100)

# Produce a static heatmap of DEGs 
gplots::heatmap.2(sig.05.res.LOA.flo_vs_bud.TMM.matrix, 
          Rowv=as.dendrogram(clustRows), 
          Colv=as.dendrogram(clustColumns),
          RowSideColors=module.color,
          col=myheatcolors2, scale='row', labRow=NA,
          density.info="none", trace="none",  
          cexRow=1, cexCol=1, margins=c(5,2),
          keysize = 1.0, key = FALSE,
          main = "Loasa heterophylla - Flower vs Bud Stage")
```

The heatmap shows that the 3737 DEGs can be clustered into two main sets whose expression is reversed between both stages.

### DEGs between stages in *Caiophora*

Now, similar steps are repeated, this time comparing *Caiophora*’s bud and flower samples.As before, the false discovery rate (FDR) threshold for significance is set to 0.05.

```
# Extract the results of the specific contrasts ----
res.CAI.flo_vs_bud <- results(dds_clc, contrast = c("sp_stage", "CAIhib_flo", "CAIhib_bud"), alpha=0.05)

summary(res.CAI.flo_vs_bud)
```

```
## 
## out of 15554 with nonzero total read count
## adjusted p-value < 0.05
## LFC > 0 (up)       : 2802, 18%
## LFC < 0 (down)     : 2502, 16%
## outliers [1]       : 182, 1.2%
## low counts [2]     : 0, 0%
## (mean count < 1)
## [1] see 'cooksCutoff' argument of ?results
## [2] see 'independentFiltering' argument of ?results
```

For this comparison, 34% of the genes are DE between bud and flower stages, again distributed about equally in up- and down-regulated genes. This represents ~10% more DEGs than the same comparison across stages for the bee-pollinated species.

An MA plot shows the log2 fold changes attributable to stage over the mean of normalized counts for all the samples. Colored points indicate DEGs.

```
# MA Plot
plotMA(res.CAI.flo_vs_bud)
```

The volcano plot shows up- (right) or down- (left) regulation on an inverted log scale so that genes showing a smaller adjusted *p-value* are higher. DEGs at the chosen level are shown in red.

```
#Volcano Plots
res.CAI.flo_vs_bud.tib <- as_tibble(res.CAI.flo_vs_bud, rownames = "geneID")

ggplot(res.CAI.flo_vs_bud.tib%>% filter(padj>=0.05)) +
  aes(y=-log10(padj), x=log2FoldChange) +
  geom_point(size=2, colour = "black", alpha=.2)+ 
  xlim (-10,10) + ylim(0,40)+
  geom_point(data=res.CAI.flo_vs_bud.tib %>% filter(padj<0.05), 
             colour = "red", alpha=.2)+
  labs(title="Caiophora hibiscifolia",
       subtitle = "Flower vs petal stage",
       caption=paste0("produced on ", Sys.time())) +
  theme_bw()
```

After defining the set of DEGs, they can be extracted and clustered to generate a heatmap of the *Caiophora* samples.

```
# Subset DE genes (padj < 0.05)
sig.05.res.CAI.flo_vs_bud.tib <- res.CAI.flo_vs_bud.tib %>% filter(padj<0.05) %>% arrange(desc(padj))

#Convert TMM expression values into matrix
sig.05.res.CAI.flo_vs_bud.TMM <- CAMac_loa_cai_TMM[,c(1,9:14)] %>% 
  filter(geneID %in% sig.05.res.CAI.flo_vs_bud.tib$geneID) 
geneIDs <- sig.05.res.CAI.flo_vs_bud.TMM$geneID

sig.05.res.CAI.flo_vs_bud.TMM.matrix <- sig.05.res.CAI.flo_vs_bud.TMM %>% 
  select(-geneID) %>% 
  as.matrix()
rownames(sig.05.res.CAI.flo_vs_bud.TMM.matrix) <- sig.05.res.CAI.flo_vs_bud.TMM$geneID

#cluster genes (rows) 
clustRows <- hclust(as.dist(1-cor(t(sig.05.res.CAI.flo_vs_bud.TMM.matrix), method="pearson")), method="complete") 

# cluster samples (columns)
clustColumns <- hclust(as.dist(1-cor(sig.05.res.CAI.flo_vs_bud.TMM.matrix, method="spearman")), method="complete") 

#Cut the resulting tree and create color vector for clusters.  
module.assign <- cutree(clustRows, k=3)

module.color <- rainbow(length(unique(module.assign)), start=0.1, end=0.9) 
module.color <- module.color[as.vector(module.assign)] 
myheatcolors2 <- colorRampPalette(colors=c("blue","white","red"))(100)

# Produce a static heatmap of DEGs 
gplots::heatmap.2(sig.05.res.CAI.flo_vs_bud.TMM.matrix, 
          Rowv=as.dendrogram(clustRows), 
          Colv=as.dendrogram(clustColumns),
          RowSideColors=module.color,
          col=myheatcolors2, scale='row', labRow=NA,
          density.info="none", trace="none",  
          cexRow=1, cexCol=1, margins=c(5,2),
          keysize = 1.0, key = FALSE,
          main = "Caiophora hibiscifolia - Flower vs Bud Stage")
```

The heatmap shows that the 5304 DEGs can be clustered into two main sets whose expression is reversed between both stages, a similar pattern to that shown by the other species.

### Differentially expressed genes across stages for both species.

```
#Convert TMM expression values into matrix
sig.05.res.flo_vs_bud.TMM <- CAMac_loa_cai_TMM %>% 
  filter(geneID %in% sig.05.res.CAI.flo_vs_bud.tib$geneID | geneID %in% sig.05.res.LOA.flo_vs_bud.tib$geneID) 

geneIDs <- sig.05.res.flo_vs_bud.TMM$geneID
sig.05.res.flo_vs_bud.TMM.matrix <- sig.05.res.flo_vs_bud.TMM %>% 
  select(-geneID) %>% 
  as.matrix()
rownames(sig.05.res.flo_vs_bud.TMM.matrix) <- sig.05.res.flo_vs_bud.TMM$geneID

#cluster genes (rows) 
clustRows <- hclust(as.dist(1-cor(t(sig.05.res.flo_vs_bud.TMM.matrix), method="pearson")), method="complete") 

# cluster samples (columns)
clustColumns <- hclust(as.dist(1-cor(sig.05.res.flo_vs_bud.TMM.matrix, method="spearman")), method="complete") 

#Cut the resulting tree and create color vector for clusters.  
module.assign <- cutree(clustRows, k=9)

module.color <- rainbow(length(unique(module.assign)), start=0.1, end=0.9) 
module.color <- module.color[as.vector(module.assign)] 
myheatcolors2 <- colorRampPalette(colors=c("blue","white","red"))(100)

# Produce a static heatmap of DEGs 
gplots::heatmap.2(sig.05.res.flo_vs_bud.TMM.matrix, 
          Rowv=as.dendrogram(clustRows), 
          Colv=as.dendrogram(clustColumns),
          RowSideColors=module.color,
          col=myheatcolors2, scale='row', labRow=NA,
          density.info="none", trace="none",  
          cexRow=1, cexCol=1, margins=c(5,2),
          keysize = 1.0, key = FALSE,
          main = "Flower vs Bud Stage DEGs")
```

Out of 15554 sets of assembled transcripts that share a hit to a *Camptotheca acuminata* gene model, contrasting flower versus bud stage petal tissues and setting the FDR at 0.05 yields 3737 DEGs for *Loasa heterophylla* and 5304 DEGs for *Caiophora hibiscifolia*.

The transcriptional profiles across all samples is less clear-cut than when analyzing separately by species, highlighting the differences between how transcription changes between stages in each of the two species.

```
#Convert TMM expression values into matrix
common_DEGS <- inner_join(sig.05.res.LOA.flo_vs_bud.TMM, sig.05.res.CAI.flo_vs_bud.TMM)
```

```
## Joining, by = "geneID"
```

```
sig.05.res.flo_vs_bud.TMM.common <- CAMac_loa_cai_TMM %>% 
  filter(geneID %in% common_DEGS$geneID) 

sig.05.res.flo_vs_bud.TMM.common.matrix <- sig.05.res.flo_vs_bud.TMM.common %>% 
  select(-geneID) %>% 
  as.matrix()
rownames(sig.05.res.flo_vs_bud.TMM.common.matrix) <- sig.05.res.flo_vs_bud.TMM.common$geneID

#cluster genes (rows) 
clustRows <- hclust(as.dist(1-cor(t(sig.05.res.flo_vs_bud.TMM.common.matrix), method="pearson")), method="complete") 

# cluster samples (columns)
clustColumns <- hclust(as.dist(1-cor(sig.05.res.flo_vs_bud.TMM.common.matrix, method="spearman")), method="complete") 

#Cut the resulting tree and create color vector for clusters.  
module.assign <- cutree(clustRows, k=9)

module.color <- rainbow(length(unique(module.assign)), start=0.1, end=0.9) 
module.color <- module.color[as.vector(module.assign)] 
myheatcolors2 <- colorRampPalette(colors=c("blue","white","red"))(100)

# Produce a static heatmap of DEGs 
gplots::heatmap.2(sig.05.res.flo_vs_bud.TMM.common.matrix, 
          Rowv=as.dendrogram(clustRows), 
          Colv=as.dendrogram(clustColumns),
          RowSideColors=module.color,
          col=myheatcolors2, scale='row', labRow=NA,
          density.info="none", trace="none",  
          cexRow=1, cexCol=1, margins=c(5,2),
          keysize = 1.0, key = FALSE,
          main = "Shared Flower vs Bud Stage DEGs")
```

A total of 1643 genes are shared across both sets of DEGs. Looking at the transcriptional profiles of this subset allows identification of DEG clusters, some showing more similar patterns of change between stages for both species (for example, the top blue and the bottom clusters), while others are more dissimilar.

### DEGs across species at similar stages

```
# 3.6 DEG across species ----
res.bud.cai_vs_loa <- results(dds_clc, contrast = c("sp_stage", "CAIhib_bud", "LOAhet_bud"), alpha=0.05)
summary(res.bud.cai_vs_loa)
```

```
## 
## out of 15554 with nonzero total read count
## adjusted p-value < 0.05
## LFC > 0 (up)       : 3756, 24%
## LFC < 0 (down)     : 3709, 24%
## outliers [1]       : 182, 1.2%
## low counts [2]     : 0, 0%
## (mean count < 1)
## [1] see 'cooksCutoff' argument of ?results
## [2] see 'independentFiltering' argument of ?results
```

```
sig.05.res.bud.cai_vs_loa <- res.bud.cai_vs_loa %>% 
  as_tibble(rownames = "geneID") %>%
  filter(padj<0.05) %>% 
  arrange(padj)

res.flo.cai_vs_loa <- results(dds_clc, contrast = c("sp_stage", "CAIhib_flo", "LOAhet_flo"), alpha=0.05)
summary(res.flo.cai_vs_loa)
```

```
## 
## out of 15554 with nonzero total read count
## adjusted p-value < 0.05
## LFC > 0 (up)       : 4659, 30%
## LFC < 0 (down)     : 4344, 28%
## outliers [1]       : 182, 1.2%
## low counts [2]     : 0, 0%
## (mean count < 1)
## [1] see 'cooksCutoff' argument of ?results
## [2] see 'independentFiltering' argument of ?results
```

```
sig.05.res.flo.cai_vs_loa <- res.flo.cai_vs_loa %>% 
  as_tibble(rownames = "geneID") %>%
  filter(padj<0.05) %>% 
  arrange(padj)
```

There are 7465 DEGs between *Loasa* and *Caiophora* at the bud stage. This is almost half of the total annotated genome. At the flower stage, there are even more DEGs: 9003. These results are consistent with the PCA plots, and are also consistent with the idea that transcriptional profiles diverge as organs develop and morphological divergence becomes more evident.

## Functional enrichment analyses using GO terms

There are several ways to approach functional analyses. Here, subsets of differentially expressed genes for wich a GO term assignment is available (through Trinotate annotation of *Camptotheca*’s gene models) are tested for enrichment. Three different approaches to detecting enrichment are used:

- Enrichment of terms within a subset of DEGs determined by an FDR threshold using a hypergeometric test
- Enrichment of genes ranking highly when sorted by significance of fold-change using a Wilcoxon’s sum-rank test
- Enrichment of genes ranking highly when sorted by fold-change value using a Wilcoxon’s sum-rank test

To perform all tests for enrichment, the GOfuncR package will be used.

### Generating a custom GO annotation table

Most packages used to test GO enrichment resource to existing annotation databases, a very handy approach when the study organism is included in the database. Unfortunately, that is usually **not** the case for most studies on non-traditional systems. Thus, before any testing can be performed, a GO annotation table specific for the reference species used must be generated and provided to the testing functions.

Thus, the first step is building this database for *Camptotheca*’s gene models, using the Trinotate annotation table. Each gene model can have several GO terms and all GO terms are stored in a single element of the CAMac annotation table. The following code, adapted from a script by Sarai H Stuart, extracts all terms and generates a table with a single GENEID<-GOTerm per row.

```
#Extract GO blastx table from Trinotate annotation table
CAMac_annot_GO <- CAMac_annot %>% 
  select(geneID, gene_ontology_BLASTX) %>% 
  filter(!(is.na(gene_ontology_BLASTX)))

#size for preallocation of memory is the length of gene_id column because we are matching 
#gene id and it's associated GO terms
nrows <- length(CAMac_annot_GO$geneID)
#choose the GO:####### pattern retain only this pattern (remove biological process text, etc.)
pattern <- 'GO:[:digit:]{7}'
#create a variable called "myList" with which to populate a list of GO terms
myList <- list()
#make the length of "myList" that is equal to the length of "nrows"; gene id list will be equivalent in 
#length to list of GO terms for each gene id
length(myList) <- length(nrows)
#create list of the gene ids
#gene ids are not included if not identified by gene name
geneNames <- character(nrows)
#there is now an output list for all of the identified gene ids
#loop through the gene_ontology_blast to identify and retain only the GO terms 
for(i in 1:nrows){
    #move through gene id column and output names into geneNames list
  geneNames[i] <- CAMac_annot_GO$geneID[i]
  
  #move through column that contains the GO terms we want to parse output items in list
  GOblastCol <- CAMac_annot_GO$gene_ontology_BLASTX[i] 
  
  #output a vector of strings that matches the GO:####### pattern from GOblastCol
  GOtermVec <- as.vector(c(stringr::str_match_all(GOblastCol, pattern)[[1]]))
  
  #populated myList with the vector of GO terms listed for each gene
  myList[[i]] <- GOtermVec
}
names(myList) <- geneNames
#pivot named list into a tidy table
CAMac_go_annotations <- data.frame(lapply(myList, "length<-", max(lengths(myList)))) %>%
  pivot_longer(1:31068, names_to = "gene", values_to = "go_id") %>%
  filter(!(is.na(go_id)))
head(CAMac_go_annotations)
```

```
## # A tibble: 6 x 2
##   gene           go_id     
##   <chr>          <chr>     
## 1 Cac_g000002.t1 GO:0012505
## 2 Cac_g000003.t1 GO:0005737
## 3 Cac_g000004.t1 GO:0042025
## 4 Cac_g000005.t1 GO:0005737
## 5 Cac_g000007.t1 GO:0005739
## 6 Cac_g000008.t1 GO:0005737
```

### Testing for term enrichment within DEG sets significant at a 0.05 FDR

This section aims to use an hypergeometric, or Fisher’s exact test to identify if any GO terms within genes whose fold-change shows an adjusted *p-value* smaller than 0.05 or less are over-represented - i.e., found more frequently than expected by a random draw from the total gene pool.

We will compare the results of contrasting between stages for *Loasa heterophylla* and *Caiophora hibiscifolia*.

```
#Test Loasa's and Caiophora's flower vs bud DEG set for enrichment
# Generate input dataframe: take all genes in matrix, then add a column to indicate if they are DE
input_hyper_LOA <- data.frame(CAMac_loa_cai_TMM %>% filter(geneID %in% CAMac_go_annotations$gene) %>% select(geneID))
input_hyper_LOA <- input_hyper_LOA %>% 
  mutate(is_candidate = if_else(geneID %in% sig.05.res.LOA.flo_vs_bud.tib$geneID,1,0))

input_hyper_CAI <- data.frame(CAMac_loa_cai_TMM %>% filter(geneID %in% CAMac_go_annotations$gene) %>% select(geneID))
input_hyper_CAI <- input_hyper_CAI %>% 
  mutate(is_candidate = if_else(geneID %in% sig.05.res.CAI.flo_vs_bud.tib$geneID,1,0))

#Run go_enrich, converting annotation to a dataframe as required by the function
#WARNING!!! This test takes a significant amount of time. To avoid running it every time, 
#the test result object is saved to an .RData file, so it can be re-read.
#
#IF THIS IS THE FIRST TIME RUNNING THE CODE IN THE CURRENT ENVIRONMENT, UNCOMMENT THE FOLLOWING FOUR LINES

#res_LOA.flo_vs_bud_hyper_bg <- go_enrich(input_hyper_LOA, test = "hyper", n_randsets = 1000, annotations = as.data.frame(CAMac_go_annotations))
#save(res_LOA.flo_vs_bud_hyper_bg, file = "res_LOA.flo_vs_bud_hyper_bg.Rdata")
#res_CAI.flo_vs_bud_hyper_bg <- go_enrich(input_hyper_CAI, test = "hyper", n_randsets = 1000, annotations = as.data.frame(CAMac_go_annotations))
#save(res_CAI.flo_vs_bud_hyper_bg, file = "res_CAI.flo_vs_bud_hyper_bg.Rdata")

load("res_LOA.flo_vs_bud_hyper_bg.Rdata")
write_tsv(res_LOA.flo_vs_bud_hyper_bg[[1]], "sig.05.res.LOA.flo_vs_bud.tib.GO_enrich.results.tsv")

load("res_CAI.flo_vs_bud_hyper_bg.Rdata")
write_tsv(res_CAI.flo_vs_bud_hyper_bg[[1]], "sig.05.res.CAI.flo_vs_bud.tib.GO_enrich.results.tsv")

#res_cai.flo_vs_bud_wilcox_bg_pvalue <- go_enrich(input_willi_cai, test = "wilcoxon", n_randsets = 1000, annotations = as.data.frame(CAMac_go_annotations))
#save(res_CAI.flo_vs_bud_wilcox_bg_pvalue, file = "res_CAI.flo_vs_bud_wilcox_bg_pvalue.Rdata")
```

#### Biological Process GO term enrichment

```
#Generate tables comparing Biological Process terms
go_table_loa <-
res_LOA.flo_vs_bud_hyper_bg[[1]] %>% as_tibble %>%
  filter(FWER_overrep<=0.05, ontology=="biological_process") %>%
  select(-raw_p_underrep,-FWER_underrep, -ontology)

gt_go_table_loa <- gt(go_table_loa) %>%
  tab_header(
    title = "Biological Process - Enriched Terms",
    subtitle = md("*Loasa heterophylla* flower vs bud")
  )

go_table_cai <-
res_CAI.flo_vs_bud_hyper_bg[[1]] %>% as_tibble %>%
  filter(FWER_overrep<=0.05, ontology=="biological_process") %>%
  select(-raw_p_underrep,-FWER_underrep, -ontology)

gt_go_table_cai <- gt(go_table_cai) %>%
  tab_header(
    title = "Biological Process - Enriched Terms",
    subtitle = md("*Caiophora hibiscifolia* flower vs bud")
  )

gt_go_table_loa
```

| Biological Process - Enriched Terms | | | |
| --- | --- | --- | --- |
| *Loasa heterophylla* flower vs bud | | | |
| node\_id | node\_name | raw\_p\_overrep | FWER\_overrep |
| --- | --- | --- | --- |
| GO:0015979 | photosynthesis | 3.421323e-08 | 0.000 |
| GO:0043604 | amide biosynthetic process | 6.661823e-07 | 0.001 |
| GO:0006412 | translation | 1.253350e-06 | 0.001 |
| GO:0043043 | peptide biosynthetic process | 2.225499e-06 | 0.001 |
| GO:0019684 | photosynthesis, light reaction | 7.184960e-06 | 0.010 |
| GO:0006091 | generation of precursor metabolites and energy | 7.618959e-06 | 0.010 |
| GO:0006518 | peptide metabolic process | 2.875880e-05 | 0.033 |

```
gt_go_table_cai
```

| Biological Process - Enriched Terms | | | |
| --- | --- | --- | --- |
| *Caiophora hibiscifolia* flower vs bud | | | |
| node\_id | node\_name | raw\_p\_overrep | FWER\_overrep |
| --- | --- | --- | --- |
| GO:0006412 | translation | 4.712211e-06 | 0.002 |
| GO:0043043 | peptide biosynthetic process | 9.438700e-06 | 0.005 |
| GO:0006518 | peptide metabolic process | 1.132187e-05 | 0.006 |

Remember that here term enrichment is tested for the subsets of DEGs, irrespective of whether they are upregulated or downregulated.

The odds ratio test result for each of *Loasa heterophylla*’s significantly enriched BP GO terms are plotted below.

```
#Plot results for Loasa
bp_gos_hyper_LOA <- res_LOA.flo_vs_bud_hyper_bg[[1]] %>% 
  filter(ontology=="biological_process", FWER_overrep <0.05) %>%
  select(node_id)
bp_gos_hyper_LOA <- bp_gos_hyper_LOA[,'node_id']
plot_anno_scores(res_LOA.flo_vs_bud_hyper_bg, bp_gos_hyper_LOA, annotations = as.data.frame(CAMac_go_annotations))
```

The odds ratio test result for each of *Caiophora hibiscifolia*’s significantly enriched BP GO terms are plotted below.

```
#Plot results
bp_gos_hyper_CAI <- res_CAI.flo_vs_bud_hyper_bg[[1]] %>% 
  filter(ontology=="biological_process", FWER_overrep <0.05) %>%
  select(node_id)
bp_gos_hyper_CAI <- bp_gos_hyper_CAI[,'node_id']
plot_anno_scores(res_CAI.flo_vs_bud_hyper_bg, bp_gos_hyper_CAI, annotations = as.data.frame(CAMac_go_annotations))
```

Neither species shows many significantly enriched BP terms. Interestingly, *Caiophora* shows fewer terms than *Loasa*, despite its larger DEG repertoire.

#### Molecular Function GO term enrichment

```
#Generate tables comparing Molecular Function terms
go_mf_table_loa <-
res_LOA.flo_vs_bud_hyper_bg[[1]] %>% as_tibble %>%
  filter(FWER_overrep<=0.05, ontology=="molecular_function") %>%
  select(-raw_p_underrep,-FWER_underrep, -ontology)

gt_go_mf_table_loa <- gt(go_mf_table_loa) %>%
  tab_header(
    title = "Molecular Function - Enriched Terms",
    subtitle = md("*Loasa heterophylla* flower vs bud")
  )

go_mf_table_cai <-
res_CAI.flo_vs_bud_hyper_bg[[1]] %>% as_tibble %>%
  filter(FWER_overrep<=0.05, ontology=="molecular_function") %>%
  select(-raw_p_underrep,-FWER_underrep, -ontology)

gt_go_mf_table_cai <- gt(go_mf_table_cai) %>%
  tab_header(
    title = "Molecular Function - Enriched Terms",
    subtitle = md("*Caiophora hibiscifolia* flower vs bud")
  )

gt_go_mf_table_loa
```

| Molecular Function - Enriched Terms | | | |
| --- | --- | --- | --- |
| *Loasa heterophylla* flower vs bud | | | |
| node\_id | node\_name | raw\_p\_overrep | FWER\_overrep |
| --- | --- | --- | --- |
| GO:0003735 | structural constituent of ribosome | 1.479965e-15 | 0.000 |
| GO:0005198 | structural molecule activity | 8.840920e-12 | 0.000 |
| GO:0019843 | rRNA binding | 2.347379e-05 | 0.017 |
| GO:0009055 | electron transfer activity | 6.135488e-05 | 0.027 |

```
gt_go_mf_table_cai
```

| Molecular Function - Enriched Terms | | | |
| --- | --- | --- | --- |
| *Caiophora hibiscifolia* flower vs bud | | | |
| node\_id | node\_name | raw\_p\_overrep | FWER\_overrep |
| --- | --- | --- | --- |
| GO:0003735 | structural constituent of ribosome | 9.847612e-13 | 0.000 |
| GO:0005198 | structural molecule activity | 4.504123e-10 | 0.000 |
| GO:0019843 | rRNA binding | 1.630214e-04 | 0.042 |

The odds ratio test result for each of *Loasa heterophylla*’s significantly enriched BP GO terms are plotted below.

```
#Plot results for Loasa
mf_gos_hyper_LOA <- res_LOA.flo_vs_bud_hyper_bg[[1]] %>% 
  filter(ontology=="molecular_function", FWER_overrep <0.05) %>%
  select(node_id)
mf_gos_hyper_LOA <- mf_gos_hyper_LOA[,'node_id']
plot_anno_scores(res_LOA.flo_vs_bud_hyper_bg, mf_gos_hyper_LOA, annotations = as.data.frame(CAMac_go_annotations))
```

The odds ratio test result for each of *Caiophora hibiscifolia*’s significantly enriched BP GO terms are plotted below.

```
#Plot results
mf_gos_hyper_CAI <- res_CAI.flo_vs_bud_hyper_bg[[1]] %>% 
  filter(ontology=="molecular_function", FWER_overrep <0.05) %>%
  select(node_id)
mf_gos_hyper_CAI <- mf_gos_hyper_CAI[,'node_id']
plot_anno_scores(res_CAI.flo_vs_bud_hyper_bg, mf_gos_hyper_CAI, annotations = as.data.frame(CAMac_go_annotations))
```

Again, neither species shows many significantly enriched MF terms. And again, *Caiophora* shows fewer terms than *Loasa*, despite its larger DEG repertoire.

#### GO term enrichment of inter-specific contrasts at the same stage

```
#Test inter-specific DEG set at bud stage for enrichment
# Generate input dataframe: take all genes in matrix, then add a column to indicate if they are DE
input_hyper_bud <- CAMac_loa_cai_TMM %>% 
  filter(geneID %in% CAMac_go_annotations$gene) %>% 
  select(geneID) %>%
  as.data.frame()

input_hyper_bud <- input_hyper_bud %>% 
  mutate(is_candidate = if_else(geneID %in% sig.05.res.bud.cai_vs_loa$geneID,1,0))

#Run go_enrich, converting annotation to a dataframe as required by the function
#WARNING!!! This test takes a significant amount of time. To avoid running it every time, 
#the test result object is saved to an .RData file, so it can be re-read.
#
#IF THIS IS THE FIRST TIME RUNNING THE CODE IN THE CURRENT ENVIRONMENT, UNCOMMENT THE FOLLOWING FOUR LINES

#res_bud_hyper_bg <- go_enrich(input_hyper_bud, test = "hyper", n_randsets = 1000, annotations = as.data.frame(CAMac_go_annotations))
#save(res_bud_hyper_bg, file = "res_bud_hyper_bg.Rdata")
load(file = "res_bud_hyper_bg.Rdata")
write_tsv(res_bud_hyper_bg[[1]], "sig.05.res.BUD.cai_vs_loa.tib.GO_enrich.results.tsv")
#Notice that no term is significantly enriched.

#IF THIS IS THE FIRST TIME RUNNING THE CODE IN THE CURRENT ENVIRONMENT, UNCOMMENT THE FOLLOWING TWO LINES
#res_flo_hyper_bg <- go_enrich(input_hyper_flo, test = "hyper", n_randsets = 1000, annotations = as.data.frame(CAMac_go_annotations))
#save(res_flo_hyper_bg, file = "res_flo_hyper_bg.Rdata")
load(file = "res_flo_hyper_bg.Rdata")
write_tsv(res_flo_hyper_bg[[1]], "sig.05.res.FLO.cai_vs_loa.tib.GO_enrich.results.tsv")

res_flo_hyper_bg[[1]] %>%
  filter(FWER_overrep <0.05) %>%
       select(-raw_p_underrep, -FWER_underrep)
```

```
##             ontology    node_id                           node_name
## 1 cellular_component GO:0009507                         chloroplast
## 2 cellular_component GO:0009579                           thylakoid
## 3 cellular_component GO:0009536                             plastid
## 4 cellular_component GO:0009534               chloroplast thylakoid
## 5 cellular_component GO:0031976                   plastid thylakoid
## 6 biological_process GO:0044283 small molecule biosynthetic process
## 7 cellular_component GO:0009526                    plastid envelope
## 8 biological_process GO:0006629             lipid metabolic process
##   raw_p_overrep FWER_overrep
## 1  1.744218e-07        0.000
## 2  5.429871e-06        0.000
## 3  1.038145e-05        0.000
## 4  1.517714e-05        0.000
## 5  1.517714e-05        0.000
## 6  5.572737e-06        0.003
## 7  2.141753e-04        0.025
## 8  5.766732e-05        0.039
```

No terms are enriched in the DEG set resulting from contrasting *Loasa* and *Caiophora* at bud stage. This is not surprising, given that this set is almost half of the genome. Interestingly though, a few terms are significant for the same comparison at the flower stage.

```
#Plot results
gos_hyper_FLO <- res_flo_hyper_bg[[1]] %>% 
  filter(FWER_overrep <0.05) %>%
  select(node_id)
gos_hyper_FLO <- gos_hyper_FLO[,'node_id']
plot_anno_scores(res_flo_hyper_bg, gos_hyper_FLO, annotations = as.data.frame(CAMac_go_annotations))
```

### Testing for term enrichment based on significance levels

The above approach uses a binary classification of genes: they are either DE or not, based on a cutoff threshold. An alternative approach is trying to identify enrichment based on a continuous variable. `GOfuncR` uses Wilcoxon’s rank-sum statistic, which ranks all genes based on this variable, and then tests which terms are associated to genes that are higher or lower in the ranking than expected if they were randomly assorted. This approach can be used with any ranking variable; we first use *p*-value.

```
# Generate input dataframe: extract genes and pvalues from DESeq results 
input_willi_loa <- res.LOA.flo_vs_bud %>% 
  as_tibble(rownames = 'geneID') %>% 
  filter(!(is.na(pvalue))) %>% 
  select (geneID, pvalue) %>% 
  as.data.frame()

input_willi_cai <- res.CAI.flo_vs_bud %>% 
  as_tibble(rownames = 'geneID') %>% 
  filter(!(is.na(pvalue))) %>% 
  select (geneID, pvalue) %>% 
  as.data.frame()


#Run Wilcoxon's sum-rank test
#WARNING!!! This test takes a significant amount of time. To avoid running it every time, 
#the test result object is saved to an .RData file, so it can be re-read.
#
#IF THIS IS THE FIRST TIME RUNNING THE CODE IN THE CURRENT ENVIRONMENT, UNCOMMENT THE FOLLOWING FOUR LINES

#res_LOA.flo_vs_bud_wilcox_bg_pvalue <- go_enrich(input_willi_loa, test = "wilcoxon", n_randsets = 1000, annotations = as.data.frame(CAMac_go_annotations))
#save(res_LOA.flo_vs_bud_wilcox_bg_pvalue, file = "res_LOA.flo_vs_bud_wilcox_bg_pvalue.Rdata")

#res_cai.flo_vs_bud_wilcox_bg_pvalue <- go_enrich(input_willi_cai, test = "wilcoxon", n_randsets = 1000, annotations = as.data.frame(CAMac_go_annotations))
#save(res_CAI.flo_vs_bud_wilcox_bg_pvalue, file = "res_CAI.flo_vs_bud_wilcox_bg_pvalue.Rdata")


load("res_LOA.flo_vs_bud_wilcox_bg_pvalue.Rdata")
write_tsv(res_LOA.flo_vs_bud_wilcox_bg_pvalue[[1]], "wilcox.LOA.flo_vs_bud_pvalue_GO_enrich.results.tsv")

load("res_CAI.flo_vs_bud_wilcox_bg_pvalue.Rdata")
write_tsv(res_CAI.flo_vs_bud_wilcox_bg_pvalue[[1]], "wilcox.LOA.flo_vs_bud_pvalue_GO_enrich.results.tsv")
```

#### Biological Process GO term enrichment

```
#Generate tables comparing Biological Process terms
go_table_loa_w <-
res_LOA.flo_vs_bud_wilcox_bg_pvalue[[1]] %>% as_tibble %>%
  filter(FWER_low_rank<=0.05, ontology=="biological_process") %>%
  select(-raw_p_high_rank ,-FWER_high_rank, -ontology)

gt_go_table_loa_w <- gt(go_table_loa_w) %>%
  tab_header(
    title = "Biological Process - Enriched Terms",
    subtitle = md("*Loasa heterophylla* flower vs bud")
  )

go_table_cai_w <-
res_CAI.flo_vs_bud_wilcox_bg_pvalue[[1]] %>% as_tibble %>%
  filter(FWER_low_rank<=0.05, ontology=="biological_process") %>%
  select(-raw_p_high_rank ,-FWER_high_rank, -ontology)

gt_go_table_cai_w <- gt(go_table_cai_w) %>%
  tab_header(
    title = "Biological Process - Enriched Terms",
    subtitle = md("*Caiophora hibiscifolia* flower vs bud")
  )

gt_go_table_loa_w
```

| Biological Process - Enriched Terms | | | |
| --- | --- | --- | --- |
| *Loasa heterophylla* flower vs bud | | | |
| node\_id | node\_name | raw\_p\_low\_rank | FWER\_low\_rank |
| --- | --- | --- | --- |
| GO:0043604 | amide biosynthetic process | 7.754861e-06 | 0.012 |
| GO:0043043 | peptide biosynthetic process | 3.012001e-06 | 0.004 |
| GO:0009768 | photosynthesis, light harvesting in photosystem I | 2.950041e-06 | 0.004 |
| GO:0019684 | photosynthesis, light reaction | 1.980208e-06 | 0.002 |
| GO:0006412 | translation | 1.425555e-06 | 0.001 |
| GO:0015979 | photosynthesis | 8.415722e-10 | 0.000 |

```
gt_go_table_cai_w
```

| Biological Process - Enriched Terms | | | |
| --- | --- | --- | --- |
| *Caiophora hibiscifolia* flower vs bud | | | |
| node\_id | node\_name | raw\_p\_low\_rank | FWER\_low\_rank |
| --- | --- | --- | --- |
| GO:0043043 | peptide biosynthetic process | 3.339381e-05 | 0.048 |
| GO:0006518 | peptide metabolic process | 2.500576e-05 | 0.032 |
| GO:0009733 | response to auxin | 2.484127e-05 | 0.032 |
| GO:0006412 | translation | 1.793196e-05 | 0.021 |

The results obtained using this method are quite similar to those based on a threshold, which is reassuring.

This time, plotting enriched terms shows violin plots with the distribution of rank-scores. Since more significant *p*-values are smaller, enriched terms have a median (white dot) well below the median score for the BP root node.

Below are the plots for *Loasa heterophylla*.

```
#Plot results
bp_gos_wilp_LOA <- res_LOA.flo_vs_bud_wilcox_bg_pvalue[[1]] %>% 
  filter(ontology=="biological_process", FWER_low_rank <0.05) %>%
  select(node_id)
bp_gos_wilp_LOA <- bp_gos_wilp_LOA[,'node_id']
plot_anno_scores(res_LOA.flo_vs_bud_wilcox_bg_pvalue, bp_gos_wilp_LOA, annotations = as.data.frame(CAMac_go_annotations))
```

And the plots for *Caiophora hibiscifolia*.

```
#Plot results
bp_gos_wilp_CAI <- res_CAI.flo_vs_bud_wilcox_bg_pvalue[[1]] %>% 
  filter(ontology=="biological_process", FWER_low_rank <0.05) %>%
  select(node_id)
bp_gos_wilp_CAI <- bp_gos_wilp_CAI[,'node_id']
plot_anno_scores(res_CAI.flo_vs_bud_wilcox_bg_pvalue, bp_gos_wilp_CAI, annotations = as.data.frame(CAMac_go_annotations))
```

#### Molecular Function GO term enrichment

```
#Generate tables comparing Molecular Function terms
go_mf_table_loa_w <-
res_LOA.flo_vs_bud_wilcox_bg_pvalue[[1]] %>% as_tibble %>%
  filter(FWER_low_rank<=0.05, ontology=="molecular_function") %>%
  select(-raw_p_high_rank ,-FWER_high_rank, -ontology)

gt_go_mf_table_loa_w <- gt(go_mf_table_loa_w) %>%
  tab_header(
    title = "Molecular Function - Enriched Terms",
    subtitle = md("*Loasa heterophylla* flower vs bud")
  )

go_mf_table_cai_w <-
res_CAI.flo_vs_bud_wilcox_bg_pvalue[[1]] %>% as_tibble %>%
  filter(FWER_low_rank<=0.05, ontology=="molecular_function") %>%
  select(-raw_p_high_rank ,-FWER_high_rank, -ontology)

gt_go_mf_table_cai_w <- gt(go_mf_table_cai_w) %>%
  tab_header(
    title = "Molecular Function - Enriched Terms",
    subtitle = md("*Caiophora hibiscifolia* flower vs bud")
  )

gt_go_mf_table_loa_w
```

| Molecular Function - Enriched Terms | | | |
| --- | --- | --- | --- |
| *Loasa heterophylla* flower vs bud | | | |
| node\_id | node\_name | raw\_p\_low\_rank | FWER\_low\_rank |
| --- | --- | --- | --- |
| GO:0019843 | rRNA binding | 5.091331e-05 | 0.027 |
| GO:0016168 | chlorophyll binding | 1.823058e-05 | 0.010 |
| GO:0005198 | structural molecule activity | 1.131523e-09 | 0.000 |
| GO:0003735 | structural constituent of ribosome | 5.697915e-14 | 0.000 |

```
gt_go_mf_table_cai_w
```

| Molecular Function - Enriched Terms | | | |
| --- | --- | --- | --- |
| *Caiophora hibiscifolia* flower vs bud | | | |
| node\_id | node\_name | raw\_p\_low\_rank | FWER\_low\_rank |
| --- | --- | --- | --- |
| GO:0005198 | structural molecule activity | 7.924546e-12 | 0 |
| GO:0003735 | structural constituent of ribosome | 2.149116e-13 | 0 |

Below are the plots for *Loasa heterophylla*.

```
#Plot results
mf_gos_wilp_LOA <- res_LOA.flo_vs_bud_wilcox_bg_pvalue[[1]] %>% 
  filter(ontology=="biological_process", FWER_low_rank <0.05) %>%
  select(node_id)
mf_gos_wilp_LOA <- mf_gos_wilp_LOA[,'node_id']
plot_anno_scores(res_LOA.flo_vs_bud_wilcox_bg_pvalue, mf_gos_wilp_LOA, annotations = as.data.frame(CAMac_go_annotations))
```

And the plots for *Caiophora hibiscifolia*.

```
#Plot results
mf_gos_wilp_CAI <- res_CAI.flo_vs_bud_wilcox_bg_pvalue[[1]] %>% 
  filter(ontology=="biological_process", FWER_low_rank <0.05) %>%
  select(node_id)
mf_gos_wilp_CAI <- mf_gos_wilp_CAI[,'node_id']
plot_anno_scores(res_CAI.flo_vs_bud_wilcox_bg_pvalue, mf_gos_wilp_CAI, annotations = as.data.frame(CAMac_go_annotations))
```

### Testing for term enrichment based on fold change

Instead of using *p*-value as a ranking variable, it is also possible to test enrichment using log2 fold-change (l2fc). This is a little different since now significant terms might be either at the top (upregulated) or bottom (downregulated) of the rank. While this seems more informative since it could show whether processes are turned on or off, it is important to remember that often times process regulation involves multiple genes, some turning on while others turn off. Thus, using l2fc as ranking variable could obscure, rather than clarify functional implications of differential gene expression.

```
# Generate input dataframe: extract genes and pvalues from DESeq results 
input_willi_loa_fc <- res.LOA.flo_vs_bud %>% 
  as_tibble(rownames = 'geneID') %>% 
  filter(!(is.na(log2FoldChange))) %>% 
  select (geneID, log2FoldChange) %>% 
  as.data.frame()

input_willi_cai_fc <- res.CAI.flo_vs_bud %>% 
  as_tibble(rownames = 'geneID') %>% 
  filter(!(is.na(log2FoldChange))) %>% 
  select (geneID, log2FoldChange) %>% 
  as.data.frame()

#Run Wilcoxon's sum-rank test
#WARNING!!! This test takes a significant amount of time. To avoid running it every time, 
#the test result object is saved to an .RData file, so it can be re-read.
#
#IF THIS IS THE FIRST TIME RUNNING THE CODE IN THE CURRENT ENVIRONMENT, UNCOMMENT THE FOLLOWING FOUR LINES

#res_LOA.flo_vs_bud_wilcox_bg_l2fc <- go_enrich(input_willi_loa_fc, test = "wilcoxon", n_randsets = 1000, annotations = as.data.frame(CAMac_go_annotations))
#save(res_LOA.flo_vs_bud_wilcox_bg_l2fc, file = "res_LOA.flo_vs_bud_wilcox_bg_l2fc.Rdata")

#res_cai.flo_vs_bud_wilcox_bg_l2fc <- go_enrich(input_willi_cai_fc, test = "wilcoxon", n_randsets = 1000, annotations = as.data.frame(CAMac_go_annotations))
#save(res_cai.flo_vs_bud_wilcox_bg_l2fc, file = "res_CAI.flo_vs_bud_wilcox_bg_l2fc.Rdata")


load("res_LOA.flo_vs_bud_wilcox_bg_l2fc.Rdata")
write_tsv(res_LOA.flo_vs_bud_wilcox_bg_l2fc[[1]], "wilcox.LOA.flo_vs_bud_l2fc_GO_enrich.results.tsv")

load("res_CAI.flo_vs_bud_wilcox_bg_l2fc.Rdata")
write_tsv(res_cai.flo_vs_bud_wilcox_bg_l2fc[[1]], "wilcox.LOA.flo_vs_bud_pvalue_GO_enrich.results.tsv")

#Generate tables comparing Biological Process terms
go_table_loa_w_fc <-
res_LOA.flo_vs_bud_wilcox_bg_l2fc[[1]] %>% as_tibble %>%
  filter(FWER_low_rank<=0.05 | FWER_high_rank<=0.05, ontology=="biological_process") %>%
  select(-ontology,-raw_p_low_rank, -raw_p_high_rank)

gt_go_table_loa_w_fc <- gt(rbind(head(go_table_loa_w_fc,20),tail(go_table_loa_w_fc,20))) %>%
  tab_header(
    title = "Biological Process -  Top and Bottom Enriched Terms",
    subtitle = md("*Loasa heterophylla* flower vs bud")
  )

go_table_cai_w_fc <-
res_cai.flo_vs_bud_wilcox_bg_l2fc[[1]] %>% as_tibble %>%
  filter(FWER_low_rank<=0.05 | FWER_high_rank<=0.05, ontology=="biological_process") %>%
  select(-ontology,-raw_p_low_rank, -raw_p_high_rank)

gt_go_table_cai_w_fc <- gt(rbind(head(go_table_cai_w_fc,20),tail(go_table_cai_w_fc,20))) %>%
  tab_header(
    title = "Biological Process - Top and Bottom 20 Enriched Terms",
    subtitle = md("*Caiophora hibiscifolia* flower vs bud")
  )

gt_go_table_loa_w_fc
```

| Biological Process - Top and Bottom Enriched Terms | | | |
| --- | --- | --- | --- |
| *Loasa heterophylla* flower vs bud | | | |
| node\_id | node\_name | FWER\_low\_rank | FWER\_high\_rank |
| --- | --- | --- | --- |
| GO:0050896 | response to stimulus | 1 | 0 |
| GO:0006950 | response to stress | 1 | 0 |
| GO:0009605 | response to external stimulus | 1 | 0 |
| GO:0044248 | cellular catabolic process | 1 | 0 |
| GO:0009057 | macromolecule catabolic process | 1 | 0 |
| GO:0030163 | protein catabolic process | 1 | 0 |
| GO:0006508 | proteolysis | 1 | 0 |
| GO:1901565 | organonitrogen compound catabolic process | 1 | 0 |
| GO:0009056 | catabolic process | 1 | 0 |
| GO:0044265 | cellular macromolecule catabolic process | 1 | 0 |
| GO:0051603 | proteolysis involved in cellular protein catabolic process | 1 | 0 |
| GO:0044257 | cellular protein catabolic process | 1 | 0 |
| GO:0044419 | interspecies interaction between organisms | 1 | 0 |
| GO:0043207 | response to external biotic stimulus | 1 | 0 |
| GO:0051707 | response to other organism | 1 | 0 |
| GO:0009607 | response to biotic stimulus | 1 | 0 |
| GO:0051716 | cellular response to stimulus | 1 | 0 |
| GO:0051641 | cellular localization | 1 | 0 |
| GO:0009628 | response to abiotic stimulus | 1 | 0 |
| GO:0007165 | signal transduction | 1 | 0 |
| GO:0006807 | nitrogen compound metabolic process | 0 | 1 |
| GO:0016556 | mRNA modification | 0 | 1 |
| GO:0034660 | ncRNA metabolic process | 0 | 1 |
| GO:0006725 | cellular aromatic compound metabolic process | 0 | 1 |
| GO:1901360 | organic cyclic compound metabolic process | 0 | 1 |
| GO:0009768 | photosynthesis, light harvesting in photosystem I | 0 | 1 |
| GO:0006518 | peptide metabolic process | 0 | 1 |
| GO:0022613 | ribonucleoprotein complex biogenesis | 0 | 1 |
| GO:0016072 | rRNA metabolic process | 0 | 1 |
| GO:1901566 | organonitrogen compound biosynthetic process | 0 | 1 |
| GO:0006364 | rRNA processing | 0 | 1 |
| GO:0006412 | translation | 0 | 1 |
| GO:0043043 | peptide biosynthetic process | 0 | 1 |
| GO:0043603 | cellular amide metabolic process | 0 | 1 |
| GO:0034470 | ncRNA processing | 0 | 1 |
| GO:0015979 | photosynthesis | 0 | 1 |
| GO:0042254 | ribosome biogenesis | 0 | 1 |
| GO:0043604 | amide biosynthetic process | 0 | 1 |
| GO:0034641 | cellular nitrogen compound metabolic process | 0 | 1 |
| GO:0009451 | RNA modification | 0 | 1 |

```
gt_go_table_cai_w_fc
```

| Biological Process - Top and Bottom 20 Enriched Terms | | | |
| --- | --- | --- | --- |
| *Caiophora hibiscifolia* flower vs bud | | | |
| node\_id | node\_name | FWER\_low\_rank | FWER\_high\_rank |
| --- | --- | --- | --- |
| GO:0050896 | response to stimulus | 1 | 0 |
| GO:1901700 | response to oxygen-containing compound | 1 | 0 |
| GO:0006950 | response to stress | 1 | 0 |
| GO:0042221 | response to chemical | 1 | 0 |
| GO:0006952 | defense response | 1 | 0 |
| GO:0006810 | transport | 1 | 0 |
| GO:0007154 | cell communication | 1 | 0 |
| GO:0051234 | establishment of localization | 1 | 0 |
| GO:0010033 | response to organic substance | 1 | 0 |
| GO:0051179 | localization | 1 | 0 |
| GO:0042493 | response to drug | 1 | 0 |
| GO:0007165 | signal transduction | 1 | 0 |
| GO:0023052 | signaling | 1 | 0 |
| GO:0010200 | response to chitin | 1 | 0 |
| GO:0001101 | response to acid chemical | 1 | 0 |
| GO:0098542 | defense response to other organism | 1 | 0 |
| GO:0006970 | response to osmotic stress | 1 | 0 |
| GO:0009628 | response to abiotic stimulus | 1 | 0 |
| GO:0009860 | pollen tube growth | 1 | 0 |
| GO:0035556 | intracellular signal transduction | 1 | 0 |
| GO:0006518 | peptide metabolic process | 0 | 1 |
| GO:0043604 | amide biosynthetic process | 0 | 1 |
| GO:0071704 | organic substance metabolic process | 0 | 1 |
| GO:0042254 | ribosome biogenesis | 0 | 1 |
| GO:0022613 | ribonucleoprotein complex biogenesis | 0 | 1 |
| GO:0006396 | RNA processing | 0 | 1 |
| GO:0043043 | peptide biosynthetic process | 0 | 1 |
| GO:0010467 | gene expression | 0 | 1 |
| GO:0006412 | translation | 0 | 1 |
| GO:0044238 | primary metabolic process | 0 | 1 |
| GO:0006807 | nitrogen compound metabolic process | 0 | 1 |
| GO:0016070 | RNA metabolic process | 0 | 1 |
| GO:1901360 | organic cyclic compound metabolic process | 0 | 1 |
| GO:0043170 | macromolecule metabolic process | 0 | 1 |
| GO:0006725 | cellular aromatic compound metabolic process | 0 | 1 |
| GO:0046483 | heterocycle metabolic process | 0 | 1 |
| GO:0006139 | nucleobase-containing compound metabolic process | 0 | 1 |
| GO:0090304 | nucleic acid metabolic process | 0 | 1 |
| GO:0034641 | cellular nitrogen compound metabolic process | 0 | 1 |
| GO:0009451 | RNA modification | 0 | 1 |

This approach yields many, many more enriched go terms (102) for *Loasa* and (225) for *Caiophora*). This is because we are no longer requiring genes to pass a significance test before being eligible; instead, we are ranking and selecting them just based on the average effect size between stages.

```
#Plot results - Top 5 up and downregulated genes
bp_gos_willi_LOA_5up5down <- c(head(go_table_loa_w_fc, 5)$node_id, tail(go_table_loa_w_fc, 5)$node_id)

rbind(head(go_table_loa_w_fc, 5),tail(go_table_loa_w_fc, 5)) %>%
  gt() %>%
  tab_header(
    title = "Biological Process - Enriched Terms",
    subtitle = md("*Loasa heterophylla* flower vs bud")
  )
```

| Biological Process - Enriched Terms | | | |
| --- | --- | --- | --- |
| *Loasa heterophylla* flower vs bud | | | |
| node\_id | node\_name | FWER\_low\_rank | FWER\_high\_rank |
| --- | --- | --- | --- |
| GO:0050896 | response to stimulus | 1 | 0 |
| GO:0006950 | response to stress | 1 | 0 |
| GO:0009605 | response to external stimulus | 1 | 0 |
| GO:0044248 | cellular catabolic process | 1 | 0 |
| GO:0009057 | macromolecule catabolic process | 1 | 0 |
| GO:0015979 | photosynthesis | 0 | 1 |
| GO:0042254 | ribosome biogenesis | 0 | 1 |
| GO:0043604 | amide biosynthetic process | 0 | 1 |
| GO:0034641 | cellular nitrogen compound metabolic process | 0 | 1 |
| GO:0009451 | RNA modification | 0 | 1 |

```
plot_anno_scores(res_LOA.flo_vs_bud_wilcox_bg_l2fc, bp_gos_willi_LOA_5up5down, annotations = as.data.frame(CAMac_go_annotations))
```

```
#Plot results - Top 5 up and downregulated genes
bp_gos_willi_CAI_5up5down <- c(head(go_table_cai_w_fc, 5)$node_id, tail(go_table_cai_w_fc, 5)$node_id)

rbind(head(go_table_cai_w_fc, 5),tail(go_table_cai_w_fc, 5)) %>% 
  gt() %>%
  tab_header(
    title = "Biological Process - Enriched Terms",
    subtitle = md("*Caiophora hibiscifolia* flower vs bud")
  )
```

| Biological Process - Enriched Terms | | | |
| --- | --- | --- | --- |
| *Caiophora hibiscifolia* flower vs bud | | | |
| node\_id | node\_name | FWER\_low\_rank | FWER\_high\_rank |
| --- | --- | --- | --- |
| GO:0050896 | response to stimulus | 1 | 0 |
| GO:1901700 | response to oxygen-containing compound | 1 | 0 |
| GO:0006950 | response to stress | 1 | 0 |
| GO:0042221 | response to chemical | 1 | 0 |
| GO:0006952 | defense response | 1 | 0 |
| GO:0046483 | heterocycle metabolic process | 0 | 1 |
| GO:0006139 | nucleobase-containing compound metabolic process | 0 | 1 |
| GO:0090304 | nucleic acid metabolic process | 0 | 1 |
| GO:0034641 | cellular nitrogen compound metabolic process | 0 | 1 |
| GO:0009451 | RNA modification | 0 | 1 |

```
plot_anno_scores(res_cai.flo_vs_bud_wilcox_bg_l2fc, bp_gos_willi_CAI_5up5down, annotations = as.data.frame(CAMac_go_annotations))
```

The terms found using the log2fc analysis are quite different from the previous analysis. This is due to the presence of large average fold changes in genes with very large intra-group variance that are not filtered out by significance testing.

#Gene Set Enrichment Analyses

```
#I generate two signatures related in the literature to cell wall lobeyness: "turgor pressure-cell wall interaction"
#and "intrinsic cell wall properties".

#I also generate a signature of genes that are related to cell ellongation

#and a signature that contains genes that are typically related to flower
#morphogenesis

#Protein names that correspond to genes in each
#signature were checked in uniprot.org

#Cell wall lobeyness proteins----

CAMac_trinotate_annot2 <- read_tsv("CAMac_trinotate_annotation_report.tsv", na = ".",) %>%
select(geneID,sprot_Top_BLASTX_hit)
```

```
## Rows: 41306 Columns: 19
```

```
## -- Column specification --------------------------------------------------------
## Delimiter: "\t"
## chr (10): geneID, transcript_id, sprot_Top_BLASTX_hit, prot_id, prot_coords,...
## lgl  (9): RNAMMER, sprot_Top_BLASTP_hit, Pfam, SignalP, TmHMM, gene_ontology...
```

```
## 
## i Use `spec()` to retrieve the full column specification for this data.
## i Specify the column types or set `show_col_types = FALSE` to quiet this message.
```

```
kin<-grep("Kinesin-like protein|KIN", CAMac_trinotate_annot2$sprot_Top_BLASTX_hit)
kin.d<-CAMac_trinotate_annot2[kin,]

rac<-grep("Rac-like GTP-binding protein|RAC", CAMac_trinotate_annot2$sprot_Top_BLASTX_hit)
rac.d<-CAMac_trinotate_annot2[rac,]

act<-grep("Actin-related protein|ARP", CAMac_trinotate_annot2$sprot_Top_BLASTX_hit)
act.d<-CAMac_trinotate_annot2[act,]

gaut<-grep("galacturonosyltransferase|GAUT", CAMac_trinotate_annot2$sprot_Top_BLASTX_hit)
gaut.d<-CAMac_trinotate_annot2[gaut,]

pme<-grep("Pectinesterase|PME", CAMac_trinotate_annot2$sprot_Top_BLASTX_hit)
pme.d<-CAMac_trinotate_annot2[pme,]

pmei<-grep("Pectinesterase inhibitor|PMEI", CAMac_trinotate_annot2$sprot_Top_BLASTX_hit)
pmei.d<-CAMac_trinotate_annot2[pmei,]

rho<-grep("Rho of plants|ROP", CAMac_trinotate_annot2$sprot_Top_BLASTX_hit)
rho.d<-CAMac_trinotate_annot2[rho,]

crib<-grep("CRIB domain-containing protein|RIC", CAMac_trinotate_annot2$sprot_Top_BLASTX_hit)
crib.d<-CAMac_trinotate_annot2[crib,]

ABP1<-grep("Auxin-binding protein 1|ABP1", CAMac_trinotate_annot2$sprot_Top_BLASTX_hit)
ABP1.d<-CAMac_trinotate_annot2[ABP1,]

PIN<-grep("Auxin efflux carrier component 1|PIN", CAMac_trinotate_annot2$sprot_Top_BLASTX_hit)
PIN.d<-CAMac_trinotate_annot2[PIN,]

CESA<-grep("Cellulose synthase|CESA", CAMac_trinotate_annot2$sprot_Top_BLASTX_hit)
CESA.d<-CAMac_trinotate_annot2[CESA,]

CLIP<-grep("CLIP-associated protein|CLASP", CAMac_trinotate_annot2$sprot_Top_BLASTX_hit)
CLIP.d<-CAMac_trinotate_annot2[CLIP,]

#Cell elongation proteins----

RGA<-grep("DELLA protein RGA|RGA", CAMac_trinotate_annot2$sprot_Top_BLASTX_hit)
RGA.d<-CAMac_trinotate_annot2[RGA,]

RGL<-grep("DELLA protein RGL|RGL", CAMac_trinotate_annot2$sprot_Top_BLASTX_hit)
RGL.d<-CAMac_trinotate_annot2[RGL,]

PIP<-grep("Aquaporin PIP|PIP", CAMac_trinotate_annot2$sprot_Top_BLASTX_hit)
PIP.d<-CAMac_trinotate_annot2[PIP,]

TIP<-grep("Aquaporin TIP|TIP", CAMac_trinotate_annot2$sprot_Top_BLASTX_hit)
TIP.d<-CAMac_trinotate_annot2[TIP,]

NIP<-grep("Aquaporin NIP|NIP", CAMac_trinotate_annot2$sprot_Top_BLASTX_hit)
NIP.d<-CAMac_trinotate_annot2[NIP,]

GASA<-grep("Gibberellin-regulated protein|GASA", CAMac_trinotate_annot2$sprot_Top_BLASTX_hit)
GASA.d<-CAMac_trinotate_annot2[GASA,]


#Flower transcription factors----

BLH9<-grep("BEL1-like homeodomain protein|BLH9", CAMac_trinotate_annot2$sprot_Top_BLASTX_hit)
BLH9.d<-CAMac_trinotate_annot2[BLH9,]

JAG<-grep("Zinc finger protein JAGGED|JAG", CAMac_trinotate_annot2$sprot_Top_BLASTX_hit)
JAG.d<-CAMac_trinotate_annot2[JAG,]

ETTIN<-grep("Auxin response factor|ETTIN", CAMac_trinotate_annot2$sprot_Top_BLASTX_hit)
ETTIN.d<-CAMac_trinotate_annot2[ETTIN,]

MADS<-grep("MADS-box protein", CAMac_trinotate_annot2$sprot_Top_BLASTX_hit)
MADS.d<-CAMac_trinotate_annot2[MADS,]

TCPs<-grep("Transcription factor TCP|TCP", CAMac_trinotate_annot2$sprot_Top_BLASTX_hit)
TCPs.d<-CAMac_trinotate_annot2[TCPs,]

#Generation of signatures for GSEA ----


#This a signature were cell wall lobeyness is not related
#to intrinsic cell wall properties but to an interplay 
#between cell wall lobeyness and turgor pressure

data.GSEA.ROP.RIP.Aux.MT<-rbind(kin.d, rac.d, act.d, rho.d, crib.d, ABP1.d, PIN.d, CESA.d,
                                CLIP.d)

#This a signature were cell wall lobeyness related
#to intrinsic cell wall properties

data.GSEA.Pectin<-rbind(gaut.d, pme.d, pmei.d)

#This a signature corresponds to proteins related to cell elongation

data.GSEA.Elongation<-rbind(RGA.d, RGL.d, PIP.d, TIP.d, NIP.d, GASA.d)

#This a signature corresponds to flower transcription factors

data.GSEA.TF.Flower<-rbind(BLH9.d, JAG.d, ETTIN.d, MADS.d, TCPs.d)


geneID.GSEA.ROP.RIP.Aux.MT<-data.GSEA.ROP.RIP.Aux.MT$geneID
geneID.GSEA.ROP.RIP.Aux.MT<-unique(geneID.GSEA.ROP.RIP.Aux.MT)

geneID.GSEA.Pectin<-data.GSEA.Pectin$geneID
geneID.GSEA.Pectin<-unique(geneID.GSEA.Pectin)

geneID.GSEA.Elongation<-data.GSEA.Elongation$geneID
geneID.GSEA.Elongation<-unique(geneID.GSEA.Elongation)

geneID.GSEA.TF.Flower<-data.GSEA.TF.Flower$geneID
geneID.GSEA.TF.Flower<-unique(geneID.GSEA.TF.Flower)


#GSEA----


#1)you have to count on a variable that allows the ranking of DEGs
#Only genes for which DGE p< 0.05.are used in this ranking,
#that is based on logfold change. 

#Loasa flo vs. bud
sig.05.res.LOA.flo_vs_bud.tib
```

```
## # A tibble: 3,737 x 7
##    geneID         baseMean log2FoldChange lfcSE  stat pvalue   padj
##    <chr>             <dbl>          <dbl> <dbl> <dbl>  <dbl>  <dbl>
##  1 Cac_g032403.t1    489.           2.53  1.01   2.51 0.0121 0.0499
##  2 Cac_g022990.t1     49.5          3.65  1.45   2.51 0.0121 0.0498
##  3 Cac_g007779.t1    198.           0.522 0.208  2.51 0.0121 0.0497
##  4 Cac_g032741.t1     16.2         -1.80  0.717 -2.51 0.0121 0.0497
##  5 Cac_g010035.t3     42.6          1.32  0.527  2.51 0.0121 0.0497
##  6 Cac_g000696.t2     59.4         -1.40  0.556 -2.51 0.0121 0.0497
##  7 Cac_g003292.t1    287.           1.18  0.470  2.51 0.0121 0.0497
##  8 Cac_g026262.t1    724.          -0.404 0.161 -2.51 0.0121 0.0497
##  9 Cac_g033921.t1    191.          -0.688 0.274 -2.51 0.0121 0.0497
## 10 Cac_g025502.t1     63.4         -1.60  0.636 -2.51 0.0120 0.0497
## # ... with 3,727 more rows
```

```
para.GSEA.Loa <- dplyr::select(sig.05.res.LOA.flo_vs_bud.tib, geneID, log2FoldChange)

# construct a named vector
para.GSEA.Loa.v <- para.GSEA.Loa$log2FoldChange
names(para.GSEA.Loa.v) <- as.character(para.GSEA.Loa$geneID)
para.GSEA.Loa.v <- sort(para.GSEA.Loa.v, decreasing = TRUE)


#Caiophora flo vs bud
sig.05.res.CAI.flo_vs_bud.tib
```

```
## # A tibble: 5,304 x 7
##    geneID         baseMean log2FoldChange lfcSE  stat pvalue   padj
##    <chr>             <dbl>          <dbl> <dbl> <dbl>  <dbl>  <dbl>
##  1 Cac_g004605.t1     37.9         -1.50  0.630 -2.38 0.0172 0.0498
##  2 Cac_g024355.t1     70.3         -0.773 0.325 -2.38 0.0172 0.0498
##  3 Cac_g004581.t1     15.6          2.50  1.05   2.38 0.0172 0.0498
##  4 Cac_g011621.t1    188.           1.67  0.702  2.38 0.0172 0.0498
##  5 Cac_g026940.t1    130.          -1.11  0.467 -2.38 0.0172 0.0497
##  6 Cac_g019650.t1    675.           0.341 0.143  2.38 0.0171 0.0497
##  7 Cac_g033619.t1    646.          -0.457 0.192 -2.38 0.0171 0.0497
##  8 Cac_g004612.t1    540.          -1.26  0.528 -2.38 0.0171 0.0497
##  9 Cac_g007541.t1     73.3         -0.996 0.418 -2.38 0.0171 0.0497
## 10 Cac_g018024.t1     91.9         -1.74  0.730 -2.38 0.0171 0.0497
## # ... with 5,294 more rows
```

```
para.GSEA.Cai <- dplyr::select(sig.05.res.CAI.flo_vs_bud.tib, geneID, log2FoldChange)

# construct a named vector
para.GSEA.Cai.v <- para.GSEA.Cai$log2FoldChange
names(para.GSEA.Cai.v) <- as.character(para.GSEA.Cai$geneID)
para.GSEA.Cai.v <- sort(para.GSEA.Cai.v, decreasing = TRUE)

#Between flowers
sig.05.res.flo.cai_vs_loa
```

```
## # A tibble: 9,003 x 7
##    geneID         baseMean log2FoldChange lfcSE  stat    pvalue      padj
##    <chr>             <dbl>          <dbl> <dbl> <dbl>     <dbl>     <dbl>
##  1 Cac_g029512.t1    5442.          -9.51 0.295 -32.2 2.05e-227 3.15e-223
##  2 Cac_g000050.t1    5905.          -5.19 0.163 -31.8 1.16e-221 8.92e-218
##  3 Cac_g021743.t1    3532.           9.16 0.291  31.4 4.25e-217 2.18e-213
##  4 Cac_g017137.t1    4203.           8.61 0.282  30.5 1.33e-204 5.10e-201
##  5 Cac_g014738.t2   13684.          -4.84 0.164 -29.5 1.24e-191 3.81e-188
##  6 Cac_g005221.t2    4032.           9.80 0.333  29.4 1.43e-190 3.66e-187
##  7 Cac_g029097.t1    3281.          10.5  0.360  29.1 1.65e-186 3.61e-183
##  8 Cac_g030153.t1    9805.          -9.93 0.344 -28.9 2.38e-183 4.57e-180
##  9 Cac_g025921.t1    4196.          10.1  0.370  27.4 8.74e-166 1.49e-162
## 10 Cac_g001293.t1    2873.          -5.58 0.209 -26.7 2.04e-157 3.14e-154
## # ... with 8,993 more rows
```

```
para.GSEA.Flor <- dplyr::select(sig.05.res.flo.cai_vs_loa, geneID, log2FoldChange)

# construct a named vector
para.GSEA.Flor.v <- para.GSEA.Flor$log2FoldChange
names(para.GSEA.Flor.v) <- as.character(para.GSEA.Flor$geneID)
para.GSEA.Flor.v <- sort(para.GSEA.Flor.v, decreasing = TRUE)


#Between buds
sig.05.res.bud.cai_vs_loa
```

```
## # A tibble: 7,465 x 7
##    geneID         baseMean log2FoldChange lfcSE  stat    pvalue      padj
##    <chr>             <dbl>          <dbl> <dbl> <dbl>     <dbl>     <dbl>
##  1 Cac_g000050.t1    5905.          -4.71 0.165 -28.5 2.47e-178 3.80e-174
##  2 Cac_g030153.t1    9805.         -12.3  0.433 -28.4 3.74e-177 2.87e-173
##  3 Cac_g014738.t2   13684.          -4.95 0.175 -28.3 1.58e-176 8.11e-173
##  4 Cac_g012351.t1    4056.          -9.97 0.382 -26.1 7.91e-150 3.04e-146
##  5 Cac_g005221.t2    4032.           9.35 0.365  25.6 1.81e-144 5.56e-141
##  6 Cac_g017137.t1    4203.           7.02 0.281  25.0 7.56e-138 1.94e-134
##  7 Cac_g001293.t1    2873.          -5.24 0.214 -24.5 3.15e-132 6.91e-129
##  8 Cac_g001303.t1     742.           7.18 0.313  23.0 6.35e-117 1.22e-113
##  9 Cac_g021093.t1    3079.         -10.8  0.482 -22.5 8.76e-112 1.50e-108
## 10 Cac_g020804.t1    1431.           7.35 0.328  22.4 2.06e-111 3.17e-108
## # ... with 7,455 more rows
```

```
para.GSEA.Bot <- dplyr::select(sig.05.res.bud.cai_vs_loa, geneID, log2FoldChange)

# construct a named vector
para.GSEA.Bot.v <- para.GSEA.Bot$log2FoldChange
names(para.GSEA.Bot.v) <- as.character(para.GSEA.Bot$geneID)
para.GSEA.Bot.v <- sort(para.GSEA.Bot.v, decreasing = TRUE)

#2)collections of signals 


#cell wall properties-turgor pressure interplat
gs_TCWI<-rep("TCWI", length(geneID.GSEA.ROP.RIP.Aux.MT))
#intrinsic cell wall properties
gs_ICWP<-rep("ICWP", length(geneID.GSEA.Pectin))
#cell elongation
gs_CE<-rep("CE", length(geneID.GSEA.Elongation))
#Flower transcription factors
gs_F.TF<-rep("F.TF", length(geneID.GSEA.TF.Flower))

gs_name<-c(gs_TCWI, gs_ICWP, gs_CE, gs_F.TF)
geneID<-c(geneID.GSEA.ROP.RIP.Aux.MT,geneID.GSEA.Pectin, geneID.GSEA.Elongation, geneID.GSEA.TF.Flower)
gs_TCWI_ICWP_CE_F.TF<-data.frame(gs_name,geneID)
names(gs_TCWI_ICWP_CE_F.TF)<-c("gs_name", "geneID")


#I do a first trial to check whether the comming script is working well
#using the first 43 genes in the list of genes rankes based on logfold
#change

#gs_top<-para.GSEA.Loa.v[1:43]
#gs_top<-names(gs_top)
#gs_top.enr<-rep("top.43.enr", 43)
#gs_top<-data.frame(gs_top.enr, gs_top)
#names(gs_top)<-c("gs_name", "geneID")

#3) Analysis

# Now that you have your msigdb collections ready, prepare your data
# grab the dataframe you made in step3 script
# Pull out just the columns corresponding to gene symbols and LogFC for at least one pairwise comparison for the enrichment analysis


# run GSEA using the 'GSEA' function from clusterProfiler

#myGSEA.prueba <- GSEA(para.GSEA.Loa.v, TERM2GENE=gs_top, verbose=FALSE, eps=0)
#myGSEA.prueba.df <- as_tibble(myGSEA.prueba@result)

#SCRIPT WORKS!

#Now I run GSEA with the four signatures I created in the previous
#steps

myGSEA.resL <- GSEA(para.GSEA.Loa.v, TERM2GENE=
                      gs_TCWI_ICWP_CE_F.TF, verbose=FALSE)
```

```
## no term enriched under specific pvalueCutoff...
```

```
myGSEA.resC <- GSEA(para.GSEA.Cai.v, TERM2GENE=
                      gs_TCWI_ICWP_CE_F.TF, verbose=FALSE)


myGSEA.resFlor <- GSEA(para.GSEA.Flor.v, TERM2GENE=
                          gs_TCWI_ICWP_CE_F.TF, verbose=FALSE)
```

```
## no term enriched under specific pvalueCutoff...
```

```
myGSEA.resBot <- GSEA(para.GSEA.Bot.v, TERM2GENE=
                         gs_TCWI_ICWP_CE_F.TF, verbose=FALSE)
```

```
## no term enriched under specific pvalueCutoff...
```

```
#GSEA calculates a sum statistic for each signature. It also
#permutes rows (genes) and calculates a null distribution of the
#enrichment score of each signature. Based on that distribution
#and on the observed enrichment value it is possible to calculate
#a p-value of the enrichment score.


#myGSEA.df <- as_tibble(myGSEA.prueba@result)
myGSEA.resC.df <- as_tibble(myGSEA.resC@result)

# create enrichment plots using the enrichplot package

#pdf(file="GSEA Caiophora3.pdf")

gseaplot2(myGSEA.resC, 
          geneSetID = c(1,2), #can choose multiple signatures to overlay in this plot. Son las filas del objeto
          pvalue_table = FALSE, #can set this to FALSE for a cleaner plot
          title = "Enrichment of lobeyness genes in C. hibiscifolia flowers") #can also turn off this title
```

```
#dev.off()
```

#Quadratic regressions of cell wall lobeyness, cell area and cell elongation

AGAINST POSITION ALONG THE PETAL MIDRIB

```
library(graphics)

data=read.table("cell data.txt",header=T)

names(data)
```

```
##  [1] "Sp"               "Stage"            "Foto"             "X1"              
##  [5] "Cell"             "Long_vert"        "Long_hor"         "Area"            
##  [9] "Mean"             "Min"              "Max"              "Perim."          
## [13] "Circ."            "AR"               "Round"            "Solidity"        
## [17] "Cell_n_mm2"       "Area_average"     "Area_average_um2"
```

```
data$Stage<-as.factor(data$Stage)
data$Sp<-as.factor(data$Sp)
data$Lobeyness<-1-data$Solidity
data$Elongation<-data$Long_vert/data$Long_hor


#the following lines are just to standardize the position of the
#SEM image along the petal midrib between 0 (basal) and 1 (apical)


#s1 = 5mm bud 
#s3 = mature flower

#l = L. heterophylla
#h = C.hibiscifolia

s1<-subset(data, Stage==1)
s1.l<-subset(s1, Sp=="loa")
s1.l$Foto.new <- (s1.l$Foto - min(s1.l$Foto)) / (max(s1.l$Foto)-min(s1.l$Foto))
s1.l$Foto.new <- 1- s1.l$Foto.new 
s1.l$Foto.cuad<-s1.l$Foto.new^2

s1.h<-subset(s1, Sp=="hib")
s1.h$Foto.new <- (s1.h$Foto - min(s1.h$Foto)) / (max(s1.h$Foto)-min(s1.h$Foto))
s1.h$Foto.new <- 1- s1.h$Foto.new
s1.h$Foto.cuad<-s1.h$Foto.new^2

s1<-rbind.data.frame(s1.l, s1.h)

s3<-subset(data, Stage==3)
s3.l<-subset(s3, Sp=="loa")
s3.l$Foto.new <- (s3.l$Foto - min(s3.l$Foto)) / (max(s3.l$Foto)-min(s3.l$Foto))
s3.l$Foto.new <- 1- s3.l$Foto.new
s3.l$Foto.cuad<-s3.l$Foto.new^2

s3.h<-subset(s3, Sp=="hib")
s3.h$Foto<-s3.h$Foto/max(s3.h$Foto)
s3.h$Foto.new <- (s3.h$Foto - min(s3.h$Foto)) / (max(s3.h$Foto)-min(s3.h$Foto))
s3.h$Foto.new <- 1- s3.h$Foto.new
s3.h$Foto.cuad<-s3.h$Foto.new^2

s3<-rbind.data.frame(s3.l, s3.h)

all<-rbind(s1, s3)


#Regressions----

#Cell lobeyness

all$Sp<-factor(all$Sp, levels=c("loa", "hib"))

Lobeyness.mod <-lm(Lobeyness ~ Foto.new*Sp + Foto.cuad*Sp + Foto.new*Stage +  Foto.cuad*Stage + Foto.new*Sp*Stage + Foto.cuad*Sp*Stage,data=all)
summary(Lobeyness.mod)
```

```
## 
## Call:
## lm(formula = Lobeyness ~ Foto.new * Sp + Foto.cuad * Sp + Foto.new * 
##     Stage + Foto.cuad * Stage + Foto.new * Sp * Stage + Foto.cuad * 
##     Sp * Stage, data = all)
## 
## Residuals:
##       Min        1Q    Median        3Q       Max 
## -0.153420 -0.027732 -0.004343  0.025161  0.197234 
## 
## Coefficients:
##                         Estimate Std. Error t value Pr(>|t|)    
## (Intercept)             0.095249   0.014256   6.681 1.06e-10 ***
## Foto.new                0.346359   0.064094   5.404 1.28e-07 ***
## Sphib                  -0.074549   0.019442  -3.835 0.000152 ***
## Foto.cuad              -0.242785   0.060231  -4.031 6.96e-05 ***
## Stage3                  0.018227   0.019233   0.948 0.343992    
## Foto.new:Sphib         -0.366550   0.089002  -4.118 4.87e-05 ***
## Sphib:Foto.cuad         0.277844   0.084577   3.285 0.001133 ** 
## Foto.new:Stage3        -0.013954   0.087588  -0.159 0.873524    
## Foto.cuad:Stage3        0.048367   0.083152   0.582 0.561199    
## Sphib:Stage3            0.003484   0.026847   0.130 0.896833    
## Foto.new:Sphib:Stage3   0.473564   0.124422   3.806 0.000169 ***
## Sphib:Foto.cuad:Stage3 -0.400959   0.119154  -3.365 0.000859 ***
## ---
## Signif. codes:  0 '***' 0.001 '**' 0.01 '*' 0.05 '.' 0.1 ' ' 1
## 
## Residual standard error: 0.04967 on 318 degrees of freedom
## Multiple R-squared:  0.7407, Adjusted R-squared:  0.7317 
## F-statistic: 82.58 on 11 and 318 DF,  p-value: < 2.2e-16
```

```
#log(Area)

LogArea.mod <-lm(log(Area) ~ Foto.new*Sp + Foto.cuad*Sp + Foto.new*Stage + Foto.cuad*Stage + Foto.new*Sp*Stage + Foto.cuad*Sp*Stage,data=all)
summary(LogArea.mod)
```

```
## 
## Call:
## lm(formula = log(Area) ~ Foto.new * Sp + Foto.cuad * Sp + Foto.new * 
##     Stage + Foto.cuad * Stage + Foto.new * Sp * Stage + Foto.cuad * 
##     Sp * Stage, data = all)
## 
## Residuals:
##      Min       1Q   Median       3Q      Max 
## -0.93061 -0.17664  0.00916  0.21684  1.06935 
## 
## Coefficients:
##                        Estimate Std. Error t value Pr(>|t|)    
## (Intercept)             7.41242    0.08926  83.043  < 2e-16 ***
## Foto.new               -2.58848    0.40130  -6.450 4.15e-10 ***
## Sphib                  -1.23556    0.12173 -10.150  < 2e-16 ***
## Foto.cuad               1.62690    0.37711   4.314 2.14e-05 ***
## Stage3                  0.49630    0.12042   4.121 4.81e-05 ***
## Foto.new:Sphib          1.82739    0.55725   3.279 0.001156 ** 
## Sphib:Foto.cuad        -0.48491    0.52955  -0.916 0.360513    
## Foto.new:Stage3         0.18261    0.54840   0.333 0.739365    
## Foto.cuad:Stage3        0.32581    0.52063   0.626 0.531886    
## Sphib:Stage3            0.44091    0.16809   2.623 0.009135 ** 
## Foto.new:Sphib:Stage3   2.60732    0.77902   3.347 0.000915 ***
## Sphib:Foto.cuad:Stage3 -3.01997    0.74604  -4.048 6.49e-05 ***
## ---
## Signif. codes:  0 '***' 0.001 '**' 0.01 '*' 0.05 '.' 0.1 ' ' 1
## 
## Residual standard error: 0.311 on 318 degrees of freedom
## Multiple R-squared:  0.7907, Adjusted R-squared:  0.7835 
## F-statistic: 109.2 on 11 and 318 DF,  p-value: < 2.2e-16
```

```
#Elongation

Elong.mod <-lm(Elongation ~ Foto.new*Sp + Foto.cuad*Sp + Foto.new*Stage + Foto.cuad*Stage + Foto.new*Sp*Stage + Foto.cuad*Sp*Stage,data=all)
summary(Elong.mod)
```

```
## 
## Call:
## lm(formula = Elongation ~ Foto.new * Sp + Foto.cuad * Sp + Foto.new * 
##     Stage + Foto.cuad * Stage + Foto.new * Sp * Stage + Foto.cuad * 
##     Sp * Stage, data = all)
## 
## Residuals:
##     Min      1Q  Median      3Q     Max 
## -4.1023 -0.7030 -0.2147  0.4766 16.4458 
## 
## Coefficients:
##                        Estimate Std. Error t value Pr(>|t|)    
## (Intercept)              5.3393     0.4516  11.822  < 2e-16 ***
## Foto.new                -6.2680     2.0305  -3.087 0.002201 ** 
## Sphib                   -2.1636     0.6159  -3.513 0.000508 ***
## Foto.cuad                2.4565     1.9081   1.287 0.198902    
## Stage3                   2.5412     0.6093   4.171 3.92e-05 ***
## Foto.new:Sphib           2.4237     2.8196   0.860 0.390674    
## Sphib:Foto.cuad         -0.3625     2.6794  -0.135 0.892482    
## Foto.new:Stage3         -9.0696     2.7748  -3.269 0.001199 ** 
## Foto.cuad:Stage3         6.6951     2.6343   2.542 0.011512 *  
## Sphib:Stage3            -3.7264     0.8505  -4.381 1.60e-05 ***
## Foto.new:Sphib:Stage3   14.5251     3.9418   3.685 0.000269 ***
## Sphib:Foto.cuad:Stage3 -10.8733     3.7749  -2.880 0.004241 ** 
## ---
## Signif. codes:  0 '***' 0.001 '**' 0.01 '*' 0.05 '.' 0.1 ' ' 1
## 
## Residual standard error: 1.573 on 318 degrees of freedom
## Multiple R-squared:  0.4692, Adjusted R-squared:  0.4509 
## F-statistic: 25.56 on 11 and 318 DF,  p-value: < 2.2e-16
```

```
#Plots----

#pdf("Cell lobeyness cuadratic regr2.pdf", height = 3.3, width=9)

par(mfrow=c(1,3))

#development of L. heterophylla

all$StageSp<- paste(all$Stage, all$Sp)
all$StageSp<- factor(all$StageSp, levels =c("1 loa", "3 loa", "1 hib", "3 hib"))

palette(c("cyan", "blue", "orange", "red"))


plot(Lobeyness~Foto.new, col=StageSp, ylab= "CL", xlab="PAMR", xlim=c(0,1), ylim=c(0,0.5), data= all)
#het 1
curve(0.095249 + x*0.346359 + x^2*-0.242785, from=min(all$Foto.new), to=max(all$Foto.new), add = TRUE, col= "cyan")
#het3
curve(0.095249 + 0.018227 + x*(0.346359-0.013954) + x^2*(-0.242785+0.048367), from=min(all$Foto.new), to=max(all$Foto.new), add = TRUE, col="blue")
#hib 1
curve(0.095249 -0.074549  + x*(0.346359 -0.366550 ) + x^2*(-0.242785+0.277844), from=min(all$Foto.new), to=max(all$Foto.new), add = TRUE, col= "orange")
#hib3
curve(0.095249 + 0.018227 -0.074549  + 0.003484  + x*(0.346359-0.013954-0.366550+ 0.473564) + x^2*(-0.242785+0.048367+0.277844-0.400959), from=min(all$Foto.new), to=max(all$Foto.new), add = TRUE, col="red")


legend(0.0, 0.5, legend=c("L. heterophylla bud", "L. heterophylla flower",
                          "C. hibiscifolia bud", "C. hibiscifolia flower"),
       col=c("cyan", "blue", "orange", "red"), lty=1, cex=0.8)

plot(log(Area)~Foto.new, col=StageSp, ylab= "log(CA)", xlab="PAMR", xlim=c(0,1), ylim=c(min(log(Area)),max(log(Area))), data= all)
#het 1
curve(7.41242 + x*-2.58848 + x^2*1.62690, from=min(all$Foto.new), to=max(all$Foto.new), add = TRUE, col= "cyan")
#het3
curve(7.41242 + 0.49630 + x*(-2.58848+0.18261) + x^2*(1.62690+0.32581), from=min(all$Foto.new), to=max(all$Foto.new), add = TRUE, col="blue")
#hib 1
curve(7.41242 -1.23556  + x*(-2.58848+1.82739) + x^2*(1.62690-0.48491), from=min(all$Foto.new), to=max(all$Foto.new), add = TRUE, col= "orange")
#hib3
curve(7.41242 + 0.49630-1.23556 +0.44091 + x*(-2.58848+1.82739+0.18261+2.60732 ) + x^2*(1.62690+0.32581-0.48491-3.01997), from=min(all$Foto.new), to=max(all$Foto.new), add = TRUE, col="red")


plot(Elongation~Foto.new, col=StageSp, ylab= "CLWR", xlab="PAMR", xlim=c(0,1), ylim=c(min(Elongation),max(Elongation)), data= all)
#het 1
curve(5.3393 + x*-6.2680 + x^2* 2.4565 , from=min(all$Foto.new), to=max(all$Foto.new), add = TRUE, col= "cyan")
#het3
curve(5.3393  + 2.5412 + x*(-6.2680-9.0696) + x^2*(2.4565+6.6951), from=min(all$Foto.new), to=max(all$Foto.new), add = TRUE, col="blue")
#hib 1
curve(5.3393  -2.1636  + x*(-6.2680+2.4237) + x^2*(2.4565-0.3625), from=min(all$Foto.new), to=max(all$Foto.new), add = TRUE, col= "orange")
#hib3
curve(5.3393 -2.1636  + 2.5412 -3.7264  + x*(-6.2680 -9.0696 +2.4237 + 14.5251  ) 
      + x^2*(2.4565 +6.6951-0.3625-10.8733), from=min(all$Foto.new), to=max(all$Foto.new), add = TRUE, col="red")
```

```
#dev.off()
```
